# The long noncoding RNA *Synage* regulates synapse stability and neuronal function in the cerebellum

**DOI:** 10.1038/s41418-021-00774-3

**Published:** 2021-03-24

**Authors:** Fei Wang, Qianqian Wang, Baowei Liu, Lisheng Mei, Sisi Ma, Shujuan Wang, Ruoyu Wang, Yan Zhang, Chaoshi Niu, Zhiqi Xiong, Yong Zheng, Zhi Zhang, Juan Shi, Xiaoyuan Song

**Affiliations:** 1 Hefei National Laboratory for Physical Sciences at the Microscale, CAS Key Laboratory of Brain Function and Disease, School of Life Sciences, Division of Life Sciences and Medicine, University of Science and Technology of China, Hefei, Anhui China; 2 National Laboratory of Medical Molecular Biology, Institute of Basic Medical Sciences, CAMS and PUMC, Beijing, China; 3 State Key Laboratory of Proteomics, Beijing Proteome Research Center, National Center for Protein Sciences (Beijing), Beijing Institute of Lifeomics, Beijing, China; 4 Graduate School of Biomedical Sciences, University of Texas MD Anderson Cancer Center and UTHealth, Houston, TX USA; 5 Stroke Center & Department of Neurology, The First Affiliated Hospital of USTC, Division of Life Sciences and Medicine, University of Science and Technology of China, Hefei, Anhui China; 6 Department of Neurosurgery, The First Affiliated Hospital of USTC, Division of Life Sciences and Medicine, University of Science and Technology of China, Hefei, Anhui China; 7 Institute of Neuroscience, State Key Laboratory of Neuroscience, CAS Center for Excellence in Brain Science and Intelligence Technology, Chinese Academy of Sciences, Shanghai, China; 8 MOE Key Laboratory for Membraneless Organelles and Cellular Dynamics, Hefei National Laboratory for Physical Sciences at the Microscale, CAS Key Laboratory of Brain Function and Disease, School of Life Sciences, Division of Life Sciences and Medicine, University of Science and Technology of China, Hefei, China

**Keywords:** RNA, Cell biology, Molecular biology, Neuroscience

## Abstract

The brain is known to express many long noncoding RNAs (lncRNAs); however, whether and how these lncRNAs function in modulating synaptic stability remains unclear. Here, we report a cerebellum highly expressed lncRNA, *Synage*, regulating synaptic stability via at least two mechanisms. One is through the function of *Synage* as a sponge for the microRNA miR-325-3p, to regulate expression of the known cerebellar synapse organizer *Cbln1*. The other function is to serve as a scaffold for organizing the assembly of the LRP1-HSP90AA1-PSD-95 complex in PF-PC synapses. Although somewhat divergent in its mature mRNA sequence, the locus encoding *Synage* is positioned adjacent to the *Cbln1* loci in mouse, rhesus macaque, and human, and *Synage* is highly expressed in the cerebella of all three species. *Synage* deletion causes a full-spectrum cerebellar ablation phenotype that proceeds from cerebellar atrophy, through neuron loss, on to synapse density reduction, synaptic vesicle loss, and finally to a reduction in synaptic activity during cerebellar development; these deficits are accompanied by motor dysfunction in adult mice, which can be rescued by AAV-mediated *Synage* overexpression from birth. Thus, our study demonstrates roles for the lncRNA *Synage* in regulating synaptic stability and function during cerebellar development.

## Introduction

Cerebellar development is an important process for regulating the onset of a variety of motor and non-motor behaviors [1]. The cerebellar cortex is composed of three layers: the molecular layer, the Purkinje cell (PC) layer, and the granule cell (GC) layer, from outermost to innermost [2]. The GC layer consists of small and densely packed excitatory granule neurons that make up the vast majority of neurons in the cerebellum and the brain [3]. The PC layer harbors the largest GABAergic inhibitory PCs, as well as Bergmann glial cells (BGCs), which are mainly located around PCs [4, 5]. The formation of mature neurons and stabilized synapses during development is a prerequisite for proper nervous system functionality, which require synaptic proteins. For instance, CBLN1, highly expressed in cerebellar GCs, is a synaptic protein crucial for organization of parallel fibers (PFs, axons of the GCs) and PCs [6, 7]. Similarly, LRP1, a postsynaptic transmembrane protein, forms a complex with postsynaptic N-methyl-D-aspartate receptors through PSD-95 (a postsynaptic density protein) to modulate synaptic transmission and synaptic plasticity [8–12]. However, much remains unclear about the mechanism of synaptic stability.

Long noncoding RNAs (lncRNAs) are transcripts longer than 200 nucleotides that do not translate into functional proteins (except in some cases forming small, potentially functional peptides). Like many protein-coding mRNAs, many lncRNAs also exhibit strong specificity in their spatio-temporal expression [13]. Many lncRNAs function in neurodevelopment. For example, the nervous system-specific lncRNAs *Evf2* and *Pinky* regulate neural development [14, 15]. The nuclear-enriched *GM12371* lncRNA is a prolific transcriptional regulator and is critical for synapse function in hippocampal neurons [16]. Nevertheless, the neurobiological functions of the overwhelming majority of lncRNAs remain enigmatic compared with protein-coding genes. Importantly, it remains unclear whether and how lncRNAs modulate synaptic stability. The *Gm2694* lncRNA (alias *AK082312*) was originally found to have enriched expression in the mouse cerebellar cortex [17]. The *Gm2694* lncRNA (alias *linc1582*) was later found to be associated with neuroectoderm differentiation [18]. Recently, *Gm2694* (alias *Trincr1*) was documented to regulate FGF/ERK signaling and the self-renewal of embryonic stem cells [19]. Although with all of these studies, whether and how *Gm2694* functions in the cerebellar synapse was mysterious.

Here, we studied this synapse-functional cerebellum lncRNA, particularly three isoforms of *Gm2694*, which we designated as *Synage*, and which is distributed in the cytoplasm and synapses of cerebellar cells. We showed that *Synage* modulates the expression of the synaptic protein CBLN1 in GCs and contributes to preventing cerebellar atrophy and motor defects. We also found that *Synage* mediates the assembly of the LRP1-HSP90AA1-PSD-95-*Synage* complex in PCs to regulate synaptic stability. Our study presents long-term in vivo data demonstrating the developmental functions of *Synage* lncRNA and reveals the mechanisms underlying its regulation of synaptic stability and cerebellar development.

## Materials and methods

### Animal models

Wild-type (WT) BALB/C and C57BL/6 mice were purchased from the Vital River Laboratories (Beijing, China) and housed in a humidity and temperature-controlled room under a reverse 12-h dark–light cycle. Mice were provided with food and water ad libitum under standard conditions. All procedures were in accordance with the guidelines of and approved by the University of Science and Technology of China Animal Resources Center and University Animal Care and Use Committee (Permit Number: USTCACUC1801015).

The sgRNAs were designed to target exon 1 and the end of last exon of *Synage*, thus excising almost the entire *Synage* locus, while avoiding the shared promoter region with *Cbln1*, to produce *Synage* knockout (KO) mice using the CRISPR-Cas9 system. Subsequently, genomic DNA was extracted and used to identify mouse genotypes with three pairs of specific primers for polymerase chain reaction (PCR) amplification and sequencing. In this study, the WT offspring of *Synage* heterozygous KO mice were used as the control group for *Synage* homozygous mice. BIOGLE Company provided F1 heterozygous KO mice. The sgRNA, shRNA, and genotyping primer sequences are listed in Supplementary Table S3.

### Reverse transcription and quantitative PCR (RT-qPCR)

Total RNAs were extracted using TRIzol reagent (Ambion, 15596) following the manufacturer’s instructions. RNase-free DNase (Promega, M6101) was used to remove residual DNA. Reverse transcription was performed using the commercially available reverse transcription system (Promega, A5001). The real-time qPCR experiments were performed on a Bio-Rad CFX96 qPCR system according to the manufacturer’s instructions (Vazyme, Q111-03). Gene expression levels were calculated relative to the reference gene *GAPDH*. The RT-qPCR primer sequences are listed in Supplementary Table S3.

### 5′- and 3′-Rapid amplification of cDNA ends (RACE)

Both 5′- and 3′-RACE experiments were performed following the manufacturer’s instructions (Invitrogen, 18374-058, 18373-019). Briefly, 5′ and 3′ gene-specific primers (GSPs) were designed to perform amplification of cDNA ends using the RACE-Ready cDNA as a template. The cDNA products were then inserted into a pRACE vector. Both PCR screening with GSPs and Sanger sequencing were further performed to identify the full-length cDNA of the target gene.

### Western blot

Mouse tissue was dissected on ice. The whole cerebellum was then transferred to a homogenizer and added to 500-μl RIPA buffer (50-mM Tris-HCl (pH 7.4), 150-mM NaCl, 1-mM EDTA, 1% Triton X-100, 1% SDS, 1% sodium-deoxycholate, fresh proteinase inhibitor cocktail, PMSF) for every 20 mg of samples. After homogenization and constant agitation at 4 °C for 2 h, each sample was centrifuged at 14,000 *g* for 10 min at 4 °C. The supernatant was aspirated and placed in a fresh tube kept on ice. The lysate was then diluted with 2X SDS loading buffer (125-mM Tris-HCl (pH 6.8), 4% SDS, 20% glycerol, 0.2% bromophenol blue, 10% β-mercaptoethanol) and boiled at 100 °C for 5 min and stored at −80 °C. The subsequent Western blot protocol was performed as described previously [20].

### Co-immunoprecipitation (co-IP)

The whole homogenized mouse cerebellum was lysed in 1-ml NET-N buffer (20-mM Tris-HCl (pH 8.0), 125-mM NaCl, 1-mM EDTA, 0.5% NP-40, 10% glycerol, fresh proteinase inhibitor cocktail, PMSF). The lysate was incubated at 4 °C for 1.5 h on a rotating platform and subsequently treated with ultrasonication. The sonicated lysate was cleared by centrifugation at 14,000 *g* for 10 min at 4 °C, then the supernatant was pre-cleared with Protein A/G Magnetic Beads (Thermo, 88803). Next, the supernatant was incubated with specific antibodies (HSP90AA1 (Proteintech, 13171-1-AP), or LRP1 (Abcam, ab92544), PSD-95 (Abcam, ab2723), or control IgG (Cell Signaling Technology, 5873/ 8726)) overnight on a rotating platform at 4 °C. Subsequently, Protein A/G Magnetic Beads were added to the supernatant for 2 h at 4 °C under gentle rotation. The beads were pelleted and washed three times with ice-cold NET-N buffer. The sample was then boiled in 1X SDS loading buffer at 100 °C for 5 min before Western blot detection.

### *Synage* overexpression and co-IP

Cells (1 × 10^6^) were transfected with *Synage* overexpression plasmid (V12-PLKO.1) and then cultured in a 6-cm dish. Forty-eight hours later, the overexpressed *Synage* cells were pelleted at 500 g for 5 min. After washing twice with phosphate-buffered saline (PBS), cell pellets were resuspended in 0.7-ml NET-N buffer with fresh proteinase inhibitor cocktail and PMSF, and incubated at 4 °C with rotation for 60 min. The lysate was isolated by centrifugation at 14,000 *g* for 10 min. The co-IP protocol was performed as described above.

### RNA immunoprecipitation (RIP)

The whole homogenized mouse cerebellum was cross-linked in 1% formaldehyde (Sangon Biotech, A501912-0500) for 10 min on a rotating platform. To stop the reaction, 0.125-M glycine was added for 10 min and the sample was pelleted at 2000 g for 5 min. Then the cell pellet was incubated in NET-N buffer for 1.5 h at 4 °C. The lysate was sonicated and centrifuged at 14,000 *g* for 10 min. Next, the supernatant was divided into two samples and incubated with specific antibodies and control IgG overnight on a rotating platform at 4 °C. Next, the supernatant was incubated with Protein A/G Magnetic Beads for 2 h at 4 °C under gentle rotation. Beads were recovered and washed three times with NET-N buffer. The beads were resuspended in proteinase K buffer (100-mM NaCl, 10-mM Tris-HCl (pH 7.0), 1-mM EDTA, 0.5% SDS, 20-μg/ml proteinase K) for 20 min at 56 °C. The remaining beads were used for RNA extraction and RT-qPCR analysis to identify interacting RNA segments according to the manufacturer’s instructions.

### In vivo RNA pull-down–mass spectrometry (MS)

The in vivo RNA pull-down–MS assay was performed as previously described [21]. Briefly, the whole homogenized mouse cerebellum was cross-linked in 1% formaldehyde for 10 min, followed by 0.125-M glycine quenching for 5 min. The sample was pelleted at 2000 g for 5 min. Cross-linked cell pellets were lysed in lysis buffer (50-mM Tris-HCl (pH 7.0), 10-mM EDTA, 1% SDS, fresh proteinase inhibitor cocktail, PMSF, Murine RNase inhibitor) and solubilized by sonication. The supernatant was incubated with biotinylated antisense oligo probes in hybridization buffer (750-mM NaCl, 1% SDS, 50-mM Tris-Cl pH 7.0, 1-mM EDTA, 15% formamide, fresh proteinase inhibitor cocktail, PMSF, Murine RNase inhibitor) at 37 °C overnight under gentle rotation. M-280 Streptavidin beads (Invitrogen, 11206D) were added to the lysis buffer and incubated at 37 °C for 30 min with rotation. The RNA-binding protein complex components were washed five times using wash buffer (2X SSC, 0.5% SDS, fresh PMSF). For protein elution, beads were resuspended in protein elution buffer (7.5-mM, N-2-hydroxyethylpiperazine-N-2-ethanesulfonic acid (HEPES, pH 7.5), 15-mM EDTA, 0.15% SDS, 75-mM NaCl, 0.02% sodium-deoxycholate) at 25 °C for 20 min and at 65 °C for 10 min. Twenty-five percent total volume TCA (trichloroacetic acid) was added to the clean eluent, and mixed proteins were precipitated at 4 °C overnight. Subsequently, proteins were pelleted at 16,000 g at 4 °C for 30 min. The supernatant was removed, and the protein pellet was washed with cold acetone and pelleted again at 16,000 g at 4 °C for 30 min. The pellet was left to air-dry for 1 min and stored at −80 °C before MS.

### Biotin-mmu-miR-325-3p pull-down

The procedure for miRNA pull-down was performed as previously described [22]. In brief, for biotin-labeled miRNA pull-down experiments, 200-pmol biotin-labeled mmu-miR-325-3p (GenePharma) were transfected into 2 × 10^6^ HT-22 cells. After 24 h, the cells were lysed in lysis buffer (20-mM Tris (pH 7.4), 100-mM KCl, 0.3% NP-40, 5-mM MgCl_2_, fresh proteinase inhibitor cocktail, PMSF, Murine RNase inhibitor). Streptavidin magnetic beads (Invitrogen, 11206D) were added to the cell lysate and incubated at 4 °C for 4 h with rotation. The M-280 Streptavidin beads were washed three times using lysis buffer. RNA bound to the M-280 magnetic beads was isolated using TRIzol LS reagent (Invitrogen, 10296028) and quantified by RT-qPCR.

### Electrophoretic mobility shift assays (EMSAs)

EMSAs were performed using the Chemiluminescent EMSA Kit according to the manufacturer’s protocol (Beyotime, GS009). Briefly, whole cerebellum tissue lysate was prepared in cytoplasmic lysis buffer (1% Triton X-100, 25-mM Tris-HCl (pH 7.4), 40-mM KCl) with fresh proteinase inhibitor cocktail, PMSF, and RNase inhibitors. The cerebellum lysate was centrifuged at 14,000 *g* for 10 min, after which the supernatant was treated with M-280 Streptavidin beads (Invitrogen, 11206D) to pre-clear the cytoplasmic extract (CE). Biotin-labeled *Synage* RNA probes (150 ng) were incubated with 14-μg CE. Unlabeled *Synage* RNA probes (7500 ng) were used as a competitor. The reaction was separated by native 6% PAGE gels and transferred onto nylon membranes. Biotin signals were detected by chemiluminescence.

### Immunofluorescence (IF)

The mice were anesthetized and intracardially perfused with PBS. The brain was dissected and fixed in 4% paraformaldehyde (PFA) overnight and dehydrated with a sucrose gradient. The frozen sagittal sections (thickness, 8–10 μm) were washed three times with 1X PBS. The sections were permeabilized by 0.1% Triton X-100 for 30 min. Tissue sections were incubated in 3% bovine serum albumin (BSA) for 1 h and then incubated with primary antibodies overnight at 4 °C. Tissue sections were washed with 1X PBS three times and subsequently incubated in secondary antibodies for 1–2 h at room temperature (RT). Tissues were washed with 1X PBS three times and incubated for 2 min with Hoechst 33342 for nuclear counterstaining. The immunostained tissues were visualized using an FV1200 confocal microscope system (OLYMPUS, Japan). The cerebellum sections with the largest area in each mouse were selected for cerebellar developmental phenotype analysis. The following primary antibodies were used: PSD-95 (Abcam, ab2723), LRP1 (Abcam, ab92544), HSP90AA1 [Alexa Fluor^®^ 647] (Novus, NBP1-77682AF647), CBLN1 (Abcam, ab181379), Calbindin-D28k (Proteintech, 14479-1-AP, 66394-1-lg), Gdf10 (Santa Cruz, sc-390046). The following secondary antibodies were used: goat anti-rabbit IgG H&L (Alexa Fluor^®^ 488, Abcam, ab150077), Alexa Fluor594-conjugated goat anti-mouse IgG (H + L) (Proteintech, SA00006-3), and goat anti-rabbit IgG H&L, F (ab′) 2 Fragment (Alexa Fluor^®^ 647 conjugate, Santa Cruz, 4414).

### In vitro transcription

The cDNA of mouse *Synage* (n424059) was cloned into pcDNA3.1 vector. The plasmid was linearized to produce a DNA template, and full-length sense or antisense RNAs were transcribed in vitro using T7 RNA polymerase (Invitrogen, 18033019) or MAXIscript SP6 Transcription Kit (Invitrogen, AM1330) in combination with biotinylated NTPs (Roche, 11685597910). In vitro transcribed RNAs were further used for FISH assays in cerebellar sections directly as described below, or for EMSAs as described above.

### Fluorescence in situ hybridization (FISH)

All solutions were prepared using RNase-free reagents and diethylpyrocarbonate-treated double deionized water. Fresh-frozen brain sections (10 μm) were fixed in 4% PFA for 10 min and washed with 1X PBS three times. Sections were digested with 1 μg/ml of proteinase K buffer for 20 min and incubated in acetylation solution for 10 min. The sections were placed in the hybridization buffer (50% formamide, 5X SSC, 0.3-mg/ml tRNA) without biotinylated probes for 5 h at RT and incubated in the hybridization solution with corresponding RNA probes (200 ng/ml) for 12–14 h at 70 °C. After hybridization, the slices were washed once in 5X SSC at 70 °C and washed twice in 0.2X SSC for 30 min at 70 °C. Sections were further incubated with streptavidin antibody and other primary antibodies for 2 h at RT. Tissue sections were washed with 1X PBS three times and subsequently incubated in secondary antibodies for 1 h at RT. Tissues were washed three times with 1X PBS and incubated with Hoechst 33342 for nuclear counterstaining, then visualized using an FV1200 confocal microscope system (OLYMPUS, Japan). The following primary antibodies were used: Streptavidin, Alexa Fluor^TM^ 555 Conjugate (Thermo Fisher, S21381), Anti-PSD-95 antibody (Abcam, ab2723), Anti-LRP1 antibody (Abcam, ab92544), and HSP90 alpha Antibody [Alexa Fluor^®^ 647] (Novus, NBP1-77682AF647). Sections were then incubated in the following secondary antibodies: goat anti-rabbit IgG H&L (Alexa Fluor^®^ 488) (Abcam, ab150077, 1:1000) and Alexa Fluor594-conjugated goat anti-mouse IgG (H + L) (Proteintech, SA00006-3, 1:500). Biotin-labeled short specific probes for *Synage* lncRNA were used in RNA-FISH assays in C8-D1A cell line, lacZ probes were used as negative controls (NCs).

### Transmission electron microscopy (TEM)

Mice were deeply anesthetized with 8% chloral hydrate, and then perfused and prefixed via the heart with 1X PBS and 4% PFA. The brain tissue was removed and immediately fixed in 4% PFA for 1–2 h at RT. The target sample (200-µm thick, 1 mm^2^) was obtained using a vibratome and disposable biopsy punches (Robbins instruments, RBP-10). The sample was fixed with 2% PFA and 3% glutaraldehyde in 0.1-M phosphate buffer and 1% osmium tetroxide in 0.1-M cacodylate buffer (pH 7.4). The sample was then dehydrated using graded ethanol, followed by embedding with EPON 812. Ultra-thin sections (60-nm thick, 200 µm^2^) were obtained and stained. Finally, electron micrographs were taken at final magnification of ×11,000 and ×30,000. All electron micrographs were analyzed by ImageJ plugins.

### Electrophysiology

Mice were deeply anesthetized with pentobarbital sodium (2% w/v, i.p.) and intracardially perfused with ~20-ml ice-cold oxygenated modified N-methyl-D-glucamine artificial cerebrospinal fluid (NMDG ACSF) that contained 93-mM N-methyl-D-glucamine (NMDG), 2.5-mM KCl, 1.2-mM NaH_2_PO_4_, 30-mM NaHCO_3_, 20-mM HEPES, 25-mM glucose, 2-mM thiourea, 5-mM Na-ascorbate, 3-mM Na-pyruvate, 0.5-mM CaCl_2_, 10-mM MgSO_4_, and 3-mM glutathione (GSH). The pH of the ACSF was 7.3–7.4, and osmolarity was 300–305 mOsm/kg. Coronal slices (250 µm) were sectioned at 0.18 mm/s on a VT1200s vibrating microtome (Leica, Germany). The brain slices were initially incubated in NMDG ACSF for 10–12 min at 33 °C, followed by HEPES ACSF that contained 92-mM NaCl, 2.5-mM KCl, 1.2-mM NaH_2_PO_4_, 30-mM NaHCO_3_, 20-mM HEPES, 25-mM glucose, 2-mM thiourea, 5-mM Na-ascorbate, 3-mM Na-pyruvate, 2-mM CaCl_2_, 2-mM MgSO_4_, and 3-mM GSH (pH 7.3–7.4, osmolarity 300–305 mOsm/kg) for at least 1 h at 28 °C.

The brain slices were transferred to a slice chamber (Warner Instruments, USA) for electrophysiological recording and were continuously perfused with standard ACSF that contained 124-mM NaCl, 2.4-mM CaCl_2_, 5-mM KCl, 1.3-mM MgSO_4_, 26.2-mM NaHCO_3_, 1.2-mM KH_2_PO_4_, and 10-mM glucose (pH: 7.3–7.4, osmolarity: 300–305 mOsm/kg) at 2.5–3 ml/min at 32 °C. The temperature of the ACSF was maintained by an in-line solution heater (TC-344B, Warner Instruments, USA). Patch pipettes (3–5 MΩ) were pulled from borosilicate glass capillaries (VitalSense Scientific Instruments Co., Ltd., Wuhan, China) with an outer diameter of 1.5 mm on a four-stage horizontal puller (P1000, Sutter Instruments, USA). For recording miniature inhibitory postsynaptic current (mIPSCs), the pipettes were filled with intracellular solution that contained 145-mM CsCl, 10-mM EGTA, 10-mM HEPES, 2-mM MgCl_2_, 2-mM CaCl_2_, 2-mM Mg-ATP, and 5-mM QX-314. The osmolarity of the solution was adjusted to 285–290 mOsm/kg, and the pH was adjusted to 7.2 with CsOH. 6,7-dinitroquinoxaline-2,3(1H,4H)-dione (10 μM) was added to eliminate excitatory components, and 1-μM tetrodotoxin (TTX) was added to the bath solution to eliminate spontaneous action potentials. For recording miniature excitatory postsynaptic current (mEPSCs), the pipettes were filled with an intracellular solution that contained 130-mM K-gluconate, 2-mM MgCl_2_, 5-mM KCl, 0.6-mM EGTA, 10-mM HEPES, 2-mM Mg-ATP, and 0.3-mM Na-GTP (osmolarity: 290–300 mOsm/kg), and the pH was adjusted to 7.4 with KOH. To abolish the inhibitory synaptic transmission, 50-μM PTX and 1-μM TTX were added in the standard ACSF. All electrophysiological recordings were Bessel-filtered at 2.8 KHz and sampled at 100 KHz. All electrophysiological data were analyzed by pCLAMP software version 10.7 (Axon Instruments, USA).

### Virus injection

AAV-EGFP-control or AAV-EF1α-*Synage* virus (BrainVTA, China) was injected into the cerebellum of randomly assigned neonatal WT and *Synage* KO mice. Briefly, neonatal mice were anesthetized on ice for 4 min and placed into a stereotaxic frame. An injecting pipette containing 300–400 nl of the AAV was injected into the cerebellum, then the mice were placed on a heating pad until they woke up. The whole procedure was completed in <20 min. The injected pups were then transferred to the mother for care after they recovered normal movement.

### Rotarod test

Rotarod tests were performed during the light phase using a rotarod training system (XR1514, Xinruan, Shanghai, China). Before the training sessions, mice were placed in the behavioral test room at least 30 min in advance in order to adapt to the environment. Mice were then habituated to stay on the stationary rod for 2 min. Subsequently, mice were placed on a rotarod apparatus that accelerates 4–40 rpm for 5 min. Most mice fell before 40 rpm. Mice were trained in three trials for 20-min intervals per day for 2 consecutive days. The rotarod was cleaned between individual tests. Latency of falling and total running distance were recorded automatically. The mice that did not move during the rotarod test were not analyzed.

### Balance beam test

The balance beam tests were performed as previously described [23]. Briefly, the beam apparatus included 120-cm beams with a flat surface of 10 or 8-mm width placed on two brackets 50 cm above the table top. On training days, each mouse crossed the center 80 cm of a 10-mm beam three times, and subsequently an 8-mm beam three times. Mice were trained in six trials in 10-min intervals per day for 2 consecutive days. On the test day, the time to cross the center 80 cm of each beam was measured and recorded. The beams were cleaned with 75% ethanol before each trial and between each mouse. The mice that did not move during the balance beam test were not analyzed.

### Quantification and statistical analysis

All experiments were independently repeated at least three times with similar results. All statistical analyses were conducted using GraphPad software. To assess the statistical significance of a difference between two treatments, we used unpaired, two-tailed Student’s *t* tests. We used two-way ANOVA followed by Tabular’s multiple comparisons for multiple groups. Statistical significances are shown as *P < 0.05, **P < 0.01, ***P < 0.001, and data are shown as the mean ± SEM.

## Results

### Conserved *Synage* lncRNA is highly expressed in the cerebellum from mouse to human

**Fig. 1:**
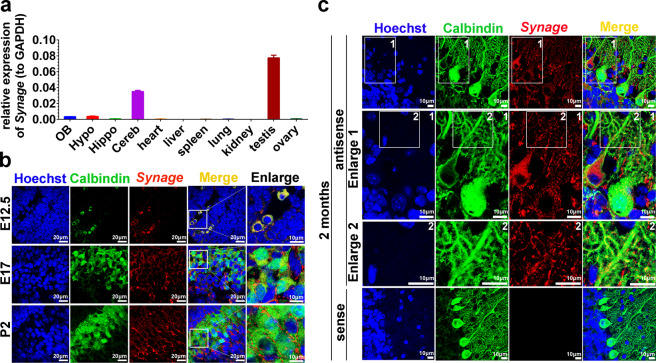
*Synage* lncRNA is mainly distributed in the cytoplasm and dendrites of cerebellar neurons. **a** Relative expression levels of *Synage* in four brain regions (OB olfactory bulb, hypo hypothalamus, hippo hippocampus, cereb cerebellum) and seven organs (heart, liver, spleen, lung, kidney, testis, and ovary) of adult mice detected by RT-qPCR. **b,** **c** RNA-FISH of *Synage* (using an antisense probe, red) and immunofluorescence of Calbindin (green, a Purkinje cell marker) in the cerebellar sections from E12.5 (embryo at 12.5 days), E17, P2 (the second day after birth) (**b**), and 2-month-old (**c**) wild-type (WT) mice. RNA-FISH using sense probe of *Synage* was a negative control. Nuclei were stained with Hoechst 33342 (blue). The numbers (1 and 2) show the enlarged areas.

To study cerebellar-relevant lncRNAs, we selected a highly expressed lncRNA, *Synage*, from our ribo-minus RNA-Sequencing (rmRNA-Seq) data in adult mouse brains (BIG Data accession number CRA001819). *Synage* is synonymous with the *Gm2694* gene, particularly three isoforms of *Gm2694*—n424059, n285242, and n264625. We examined the abundance and tissue specificity of *Synage* transcript across different organs and brain regions by RT-qPCR. The results showed that *Synage* was specifically enriched in the cerebellum and testis of adult mice (Fig. 1a). In the current study, we focused on the function of *Synage* in the cerebellum.

We first performed RNA-FISH using a biotin-labeled *Synage* antisense RNA probe in the C8-D1A cell line (astrocyte type I cloned cell line from 8-day postnatal mouse cerebella [24]) and mouse cerebellum sections at several developmental stages, including E12.5, E17, P2, and 2 months. *Synage* was mainly distributed in the cytoplasm of C8-D1A cerebellum cells (Fig. S1), and was specifically distributed in the Purkinje cell precursors (PCPs) at E12.5, while at E17, P2, and 2 months, it was localized primarily in cytoplasm and dendrites of cerebellar cells, including PCs (Fig. 1b, c).

*Synage*-homologous genes (*LOC106995009* in rhesus macaque, and *RP11-491F9.1* in human) were conserved in terms of their locations in the genomes of mouse, rhesus macaque, and human (adjacent to the *Cbln1* gene) (Fig. S2a–c). Similar to *Synage* lncRNA in mouse [17], its homologous lncRNAs exhibited cerebellum-specific expression in rhesus macaque [25] and in human tissues according to a recent study [26] and GTEx project database (dbGaP Accession phs000424.v8.p2) (Fig. S2d). Taken together, *Synage* lncRNA is conserved in its genomic location (adjacent to the *Cbln1* gene) and in its distribution specificity in the cerebellum among mouse, rhesus macaque, and human.

According to our rmRNA-Seq in mouse brains (Fig. S3a), the expression level of n424059 (one of the *Synage* isoforms) was the highest, followed by n285242 (one of the *Synage* isoforms) and TCONS_00072254 (Fig. S3b). Although the expression trend of *Synage* isoforms detected by RT-qPCR was not completely consistent with that of rmRNA-Seq (Fig. S3c–g), n424059 and n285242 were relatively highly expressed in both rmRNA-Seq and RT-qPCR. In addition, the 5′- and 3′-RACE experiments using n424059-specific primers (Fig. S4a–d) revealed that *Synage* is 663 nt in length, consistent with the length of n424059 and with cDNA sequencing conducted in this study (Fig. S4e). Therefore, our data support that n424059 is a typical sequence for *Synage* RNA; we thus used the n424059 lncRNA sequence for our following in vitro and in vivo overexpression experiments.

### *Synage* KO mice show significant cerebellar atrophy and neuronal loss during cerebellar development

**Fig. 2:**
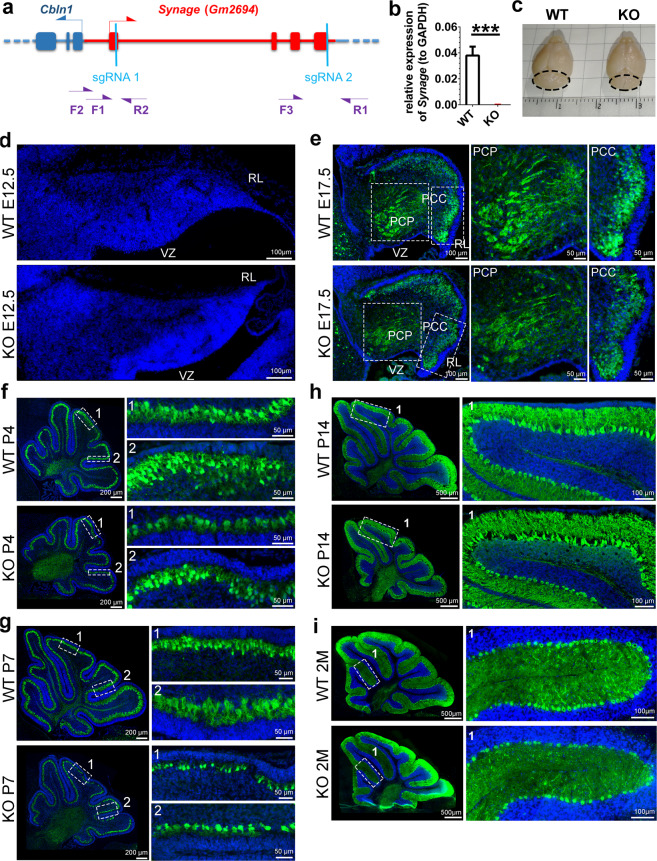
*Synage* knockout mice show cerebellar defects and neuronal loss during cerebellar development. **a** Schematic representation of the position of *Synage*, *Cbln1*, sgRNAs, and genotyping primers (F forward primers, R reverse primers). **b** Relative expression levels of *Synage* in the cerebella of WT and knockout (KO) adult mice detected by RT-qPCR. **c** Gross morphology of representative brains from adult WT and KO male mice. **d** Hoechst 33342 staining of cerebella from E12.5 WT and KO mice (WT, *n* = 9; KO, *n* = 6). **e**–**i** Immunofluorescence staining of the Purkinje cell marker protein (Calbindin, green) in the cerebella from E17.5 (**e**), P4 (**f**), P7 (**g**), P14 (**h**), and 2-month-old (**i**) WT and KO mice. WT (E17.5), *n* = 7; KO (E17.5), *n* = 3; WT (P4, P7, P14, and 2 months), *n* = 3; KO (P4, P7, P14, and 2 months), *n* = 3. Nuclei were stained with Hoechst 33342 (blue).

We generated *Synage* KO mice with sgRNAs targeting *Synage* exon 1 and the 3′ end of the last exon coupled with the CRISPR-Cas9 system, ablating most *Synage* locus, while avoiding deletion of the putative promoter region shared with *Cbln1* (Fig. 2a). F1 mice that carried heterozygous (HT) alleles were confirmed by PCR-based genotyping (Fig. S5a). Homozygous mice were obtained by crossing these HT mice, and again confirmed by PCR (Fig. S5a) and sequencing (Fig. S5b). We used RT-qPCR (Fig. 2b) and FISH (Fig. S5c) to verify the absence of the *Synage* transcript in *Synage* KO mice compared with WT mice.

Between WT and *Synage* KO mice, there were no significant changes in the body appearance (Fig. S5d), body weight (Fig. S5e, f), or brain weight (Fig. S5g, h). However, the weight of cerebella relative to body weight was significantly decreased in both female and male KO mice (Figs. S5i–k and 2c). We performed IF staining for PCs (using Calbindin, a specific marker for PCs [27–29]) on cerebellar sections of 2-month-old mice. The number of PCs in adult KO mice was significantly decreased compared to those of both WT mice and HT mice, while it did not differ between WT and HT mice (Fig. S5l, m). The protein expression levels of NeuN (a neuronal marker) and Gephyrin (an inhibitory postsynaptic marker) were also substantially reduced in *Synage* KO cerebella compared to WT (Fig. S5n, o).

Cerebellar neurons are generated from two distinct neuroepithelial zones: the ventricular zone (VZ) and the more dorsally located rhombic lip (RL) [30, 31], while PCs are differentiated/generated from the VZ at E10.5–E12.5 [32–34], before subsequently migrating dorsally to form a multi-cell-thick immature PC layer called the cerebellar plate at ∼E14.5 [35]. We further sectioned cerebella sagittally and IF stained them for PCs [27–29] at six developmental stages (E12.5, E17.5, P4, P7, P14, and 2 months) [36]. The results showed that the VZ became thinner and revealed RL invagination in E12.5 *Synage* KO mice compared to the WT controls (Fig. 2d).

Given that the PCPs expand in concert with cerebellar development, and considering that several PC clusters (PCCs) are known to aggregate with the PCs forming a multilayer below the external germinal layer by E17.5 [37], it was informative when we observed obviously reduced numbers of PCPs and of PCCs in E17.5 *Synage* KO mice compared to the WT mice (Fig. 2e). During differentiation, mouse PCs are in cluster stage around E18 to P3 [38], and are in dispersal situation at P3–P7. Premature PCs arborize (maturation process) at P12–P13; the PCs maturation ends at around P18–P20 [39], and the cerebellum undergoes dramatic increases in size and changes after birth [40]. We found that the number of PCs was reduced in *Synage* KO mice at P4 (Fig. 2f), P7 (Fig. 2g), P14 (Fig. 2h), and 2 months (Fig. 2i).

**Fig. 3:**
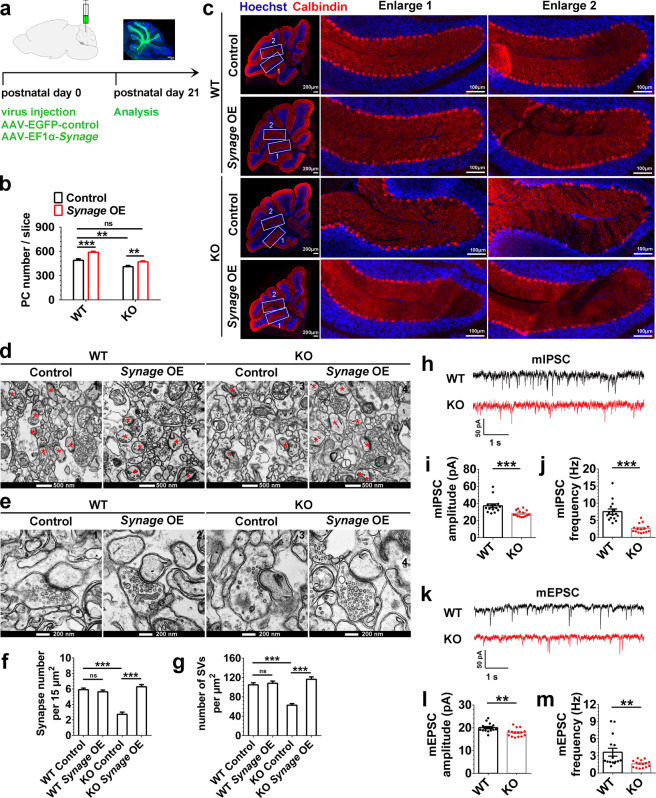
Overexpression of *Synage* in the cerebella of knockout mice rescues the numbers of both Purkinje cells and synapses to the level of wild-type mouse. **a** Schematic representation of the AAV injection (AAV-EGFP-control and AAV-EF1α-*Synage*) and the analysis strategy for WT and KO mice. **b**, **c** Quantification of the number of Purkinje cells (PCs) per cerebellar sections (**b**) and representative immunofluorescence staining images of PCs (labeled with Calbindin, red) (**c**) in 3-week-old WT and KO mouse cerebella after stereotaxic injection of AAV-EGFP-control (control) or AAV-EF1α-*Synage* (*Synage* OE) into the neonatal mouse cerebella. Nuclei were stained with Hoechst 33342 (blue). The numbers (1 and 2) show the enlarged areas. Left scale bar: 200 μm; scale bar of the enlarged regions: 100 μm (WT Control, *n* = 7; WT *Synage* OE, *n* = 8; KO Control, *n* = 14; KO *Synage* OE, *n* = 11). **d**–**e** Representative transmission electron microscopy (TEM) micrographs of synapses upon AVV-mediated *Synage* overexpression (OE) on cerebellar cortex in adult WT and *Synage* KO mice. Synapses are indicated by red asterisks, upper scale bar: 500 nm (**d**), lower scale bar: 200 nm (**e**). **f** Quantification of the number of synapses per 15 μm^2^ in three mice (WT Control, *n* = 93; WT *Synage* OE, *n* = 47; KO Control, *n* = 29; KO *Synage* OE, *n* = 53). **g** Quantification of the number of SVs per μm^2^ in WT and KO mice (WT Control, *n* = 191; WT *Synage* OE, *n* = 84; KO Control, *n* = 70; KO *Synage* OE, *n* = 74). **h**–**j** Representative traces of mIPSCs from cerebellar PCs (**h**) and quantification of mIPSC amplitude (**i**) and frequency (**j**) in P26 WT and KO cerebella. Dots indicate data from individual experiments. **k**–**m** Representative traces of mEPSCs from cerebellar PCs (**k**) and quantification of mEPSC amplitude (**l**) and frequency (**m**) in P26 WT and KO cerebella. Dots indicate data from individual experiments.

In addition, we counted the numbers of major cerebellar neurons (PCs and GCs) and BGCs in the cerebellum at P10, P23, and 2 months, using Calbindin-positive cells to represent PCs, Gdf10-stained cells to determine BGCs [41–43], and the signal intensity of Hoechst 33342 staining in the GC layer to estimate GCs (since the signals for GCs-specific marker Pax6 had too much overlay with each other to accurately quantify). The numbers of PCs and GCs, but not BGCs, significantly decreased at P10 (Fig. S6a–d), P23 (Fig. S6a, e–g), and 2 months (Fig. S6a, h–j) in *Synage* KO mice compared with WT mice. Overexpression of *Synage* in the cerebellum rescued the number of PCs to the level of WT mice at 3 weeks after injection by injecting AAV-EF1α-*Synage* into the cerebella of newborn *Synage* KO mice (Fig. 3a–c). These results supported that *Synage* is necessary for cerebellar development and maturation.

### *Synage* deletion leads to severe morphological and functional defects in synapses

Since synapses can form between neurons and are essential to neuronal function, the loss of neurons (PCs and GCs) upon *Synage* KO led us to investigate if there was also an ablation of cerebellar synapses after *Synage* KO. We performed TEM analyses on cerebellar cortex slides in adult WT and *Synage* KO mice to observe the numbers of synapses and synaptic vesicles (SVs). We found: (1) significantly reduced numbers of both synapses and SVs in presynaptic terminals in the cerebellar cortex of KO compared to WT mice (Fig. 3d–g). (2) Cerebella overexpressing *Synage* in adult WT mice showed no significant change in the number of synapses or SVs compared to WT mice (Fig. 3d–g). (3) Complementation of *Synage* expression in *Synage* KO mice rescued the numbers of both synapses and SVs (which reached the levels detected for WT mice) (Fig. 3d–g). The reduction in synapse density in *Synage* KO mice further suggests an additional deficit in synaptogenesis and/or in synapse maintenance. Moreover, the reduction in the number of SVs also shows a deficit in the structure and function of individual synapses.

The severe morphological defects in the cerebellar synapses of *Synage* KO mice suggested that synaptic connectivity and function are potentially adversely affected. We thus evaluated the excitatory and inhibitory synapses of PCs by measuring mIPSCs and mEPSCs from PCs on cerebellar sagittal slices using a whole-cell patch-clamp. Both amplitude and frequency of mIPSCs were reduced in *Synage* KO mice (Fig. 3h–j), as were mEPSCs (Fig. 3k–m). These results indicate that synaptic connectivity and function in PCs in the cerebella of *Synage* KO mice are severely compromised.

Aberrant cerebellar morphology often leads to motor behavior defects [44]. The rotarod test and the balance beam test, well-established methods to evaluate motor coordination in rodents [23, 45], showed that motor abilities and motor-dependent learning and memory were severely impaired in *Synage* KO mice (Fig. S7a, b). Taken together, our findings of the decrease in cerebellar neurons and synapses and the defects in neuronal synaptic function in *Synage* KO mice all strongly suggest that the severe morphological and functional defects in neurons and synapses are responsible for the observed motor dysfunction of *Synage* KO mice.

### *Synage* lncRNA maintains stability and function of cerebellar synapses partially by regulating *Cbln1* mRNA

*Cbln1*, a cerebellum highly expressed and synapse-related glycoprotein-coding gene, is upstream of *Synage* and is transcribed in the opposite direction (Fig. S2a). CBLN1 protein is secreted from cerebellar GCs to act as a critical synapse organizer between PFs and PCs [6, 7]. Since many lncRNAs regulate their neighboring protein-coding genes, we asked whether *Synage* lncRNA also modulates *Cbln1* expression. C8-D1A cells have poor transfection efficiency, which prevented us from performing in vitro experiments in this cell line. After screening for many cell lines, we found that two isoforms of *Synage* (n285242, n264625) were robustly expressed in the HT-22 cell line (Fig. S8a), which is a mouse hippocampal neuronal cell line that has higher transfection efficiency than the C8-D1A cell line [46, 47]. Therefore, we designed two shRNAs specifically targeting *Synage*, and transfected them into the HT-22 cell line to knockdown *Synage* (Fig. S8b). The results indicated that the expression levels of both *Cbln1* mRNA and protein were significantly reduced upon *Synage* knockdown in the HT-22 cell line (Fig. S8b–d).

**Fig. 4:**
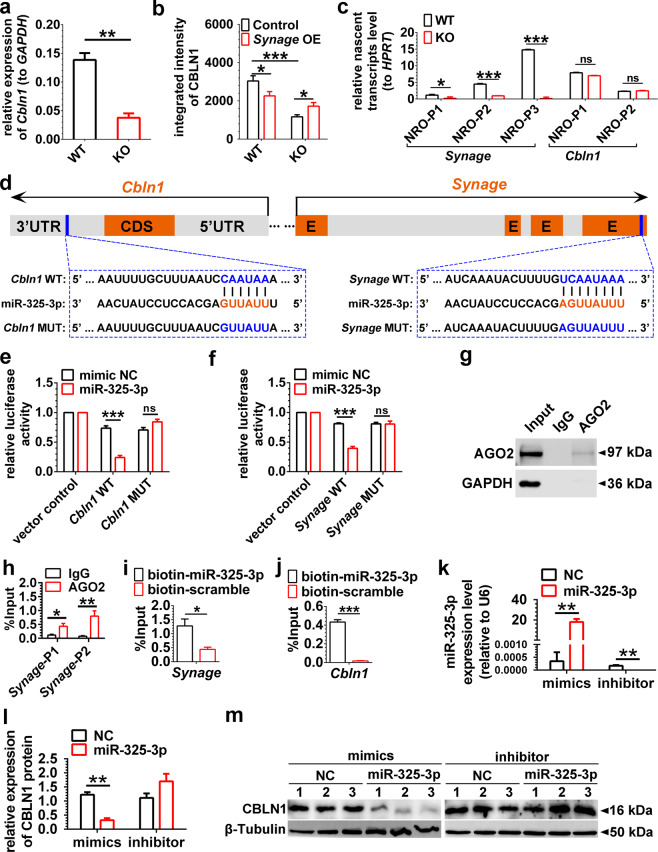
*Synage* lncRNA regulates *Cbln1* mRNA through the AGO2-miR-325-3p pathway. **a** Relative expression level of *Cbln1* mRNA in adult WT and KO mouse cerebella. **b** Relative quantification of integrated intensity of CBLN1 protein in the cerebellar cortex of 3-week-old WT and KO mice detected by immunofluorescence staining after stereotaxic injection of AAV-control or AAV-*Synage* (*Synage* OE) into the neonatal mouse cerebellum. **c** Relative nascent transcript levels of both *Synage* and *Cbln1* using multiple pairs of primers, normalized to housekeeping gene *HPRT* (hypoxanthine guanine phosphoribosyl transferase). NRO nuclear run on, P primer pairs. **d** Schematic representation of the predicted binding sites of miR-325-3p on *Synage* and *Cbln1* (CDS coding sequence, UTR untranslated region, E exon). **e** Relative luciferase activity of vector control and WT *Cbln1*-3′UTR and mutant *Cbln1*-3′UTR luciferase reporter gene plasmids upon co-transfection of mimic negative control (NC) or miR-325-3p in the HT-22 cell line. **f** Relative luciferase activity of vector control and WT *Synage* and mutant *Synage* luciferase reporter gene plasmids upon co-transfection of mimic NC or miR-325-3p in the HT-22 cell line. Data are presented as the relative ratio of firefly luciferase activity to *Renilla* luciferase activity. **g** Western blot analysis of AGO2 protein immunoprecipitation by AGO2 antibody in AGO2-RNA immunoprecipitation. **h** The amount of *Synage* lncRNA binding to AGO2 or IgG was quantified as a percentage of input in IP by RT-qPCR (*Synage*-P1, a primer pair specifically targeting n264625; *Synage*-P2, a primer pair targeting both n264625 and n285242). **i**, **j** The amount of *Synage* lncRNA (**i**) and *Cbln1* mRNA (**j**) binding to miR-325-3p was quantified as a percentage of input in miR-325-3p pull-down assays by RT-qPCR. **k–m** Relative expression levels of miR-325-3p with in vitro transfection of miR-325-3p mimics and inhibitor in the HT-22 cell line, normalized to *U6* (**k**). Quantification (**l**) and representative images of Western blots (**m**) for CBLN1 with in vitro transfection of miR-325-3p mimics and inhibitor in the HT-22 cell line.

To further test the effect of *Synage* knockdown on *Cbln1* in vivo, we injected the *Synage* shRNAs into the cerebella of adult WT mice. Two weeks later, we again observed that *Cbln1* expression was diminished in the cerebellum compared with its expression in the olfactory bulb (Fig. S8e), *Cbln1* mRNA and protein levels had a similar decrease in *Synage* KO mouse cerebellum (Figs. 4a and S8f, g). We also found that overexpression of *Synage* in its KO mouse significantly increased the CBLN1 expression compared with KO control group (Figs. 4b and S8h). Thus, both in vivo and in vitro *Synage* knockdown as well as in vivo *Synage* KO experiments demonstrated that *Synage* regulates the expression of *Cbln1*. Given the important role of *Cbln1* in the regulation of cerebellar synaptic function, these results suggest that the *Synage* lncRNA may be also involved in regulating cerebellar synaptic functions.

Given their spatial proximity in the genome, we asked whether *Synage* KO inhibiting *Cbln1* expression is due to inadvertent excision of some potential regulatory elements upstream of the *Cbln1* gene. We thus performed a nuclear run on assay [48–50] on WT and KO mice cerebella, and found that *Synage* deletion did not affect the number of nascent *Cbln1* transcripts (Fig. 4c), although the total *Cbln1* mRNA and protein levels were significantly decreased in the cerebella of 2-month-old *Synage* KO mice (Figs. 4a and S8f, g). These data suggest that *Synage* regulates *Cbln1* expression at the mRNA and/or protein levels.

*Synage* deletion exerted a strong influence on both the mRNA and protein levels of *Cbln1*; however, we did not detect the CBLN1 protein in our in vivo RNA pull-down–MS experiment as a potential *Synage*-associating protein. Considering our finding that *Synage* is localized in the cytoplasm of cerebellar cells, we explored the possibility that *Synage* may function as a ceRNA by competing with miRNAs [51, 52]. Specifically, we predicted the shared miRNA targets for *Cbln1* and *Synage* using the miRNA-target (mRNA/lncRNA) interaction modules of both StarBase v3 [53, 54] and DIANA-LncBase v2 [55], which identified a perfectly conserved seed match, mmu-miR-325-3p, in the 3′UTR region of *Cbln1* and the last exon of two isoforms of *Synage* (n285242, n264625) (Fig. 4d).

To confirm the prediction for their targeting relationship, we constructed mutant *Cbln1*-3′UTR and mutant *Synage* luciferase reporter plasmids, which retained the appropriate AT composition (Fig. 4d). The n285242 is one of the *Synage* isoforms and its full length was used in this experiment. HT-22 cells were then co-transfected with the NC or mmu-miR-325-3p and WT *Cbln1*-3′UTR or mutant *Cbln1*-3′UTR, as well as WT *Synage* or mutant *Synage* luciferase reporter gene plasmids. Dual luciferase reporter assay results showed that mmu-miR-325-3p significantly reduced the luciferase activities from both the WT *Cbln1* and WT *Synage* luciferase reporter gene plasmids; no reductions were observed with the mutant *Cbln1* or mutant *Synage* luciferase reporter gene plasmids (Fig. 4e, f). These results together indicated that mmu-miR-325-3p directly binds to the 3′UTR of *Cbln1* mRNA and to the last exon of *Synage*.

MicroRNAs silence gene expression by repressing translation and promoting target mRNA degradation [56]. To determine whether *Synage* regulates *Cbln1* as a ceRNA by competing for the above identified shared miRNA (mmu-miR-325-3p) in the AGO2-miRNA pathway, we made use of the published AGO2 CLIP-Seq data from mouse cortex tissue (GSE73058) to identify AGO2-bound RNAs [57]. We found that AGO2 had multiple binding sites located in the *Cbln1* and *Synage* (Supplementary Table S1). We performed an AGO2-RIP-qPCR experiment using mouse cerebellum tissue, which showed that n285242 and n264625 (two isoforms of *Synage*) were pulled down in the AGO2 complex (Fig. 4g, h). Conversely, a biotin-labeled mmu-miR-325-3p RNA was able to pull-down both *Synage* (by a primer pair that targeting all of the three isoforms) and *Cbln1* transcripts (Fig. 4i, j). We next determined the effects of mmu-miR-325-3p on *Synage* and *Cbln1* expression, by detecting the respective decrease or increase in CBLN1 protein in response to the mmu-miR-325-3p mimics or inhibitor in the transfected HT-22 cell line (Fig. 4k–m). Together, our data indicate that *Synage* (particularly, isoforms of n285242 and n264625) acts as a sponge for mmu-miR-325-3p to regulate *Cbln1* mRNA expression, which leads to the change of the CBLN1 protein levels.

*Cbln1*^−/−^ mice showed cerebellar ataxia and impaired performance accompanied by a significant reduction in the number of PF-PC synapses, as well as severe impairment to synaptic function (Supplementary Table S2) [6, 58–62]. *Synage* KO mice exhibited phenotypes consistent with these reports, including synapse reduction and dysfunction, as well as motor defects, but otherwise showed more severe impairment than the phenotypes of *Cbln1*^−/−^ mice, including decreased SVs, obvious neuronal loss, decreased cerebellar weight, and reduced fertility (Supplementary Table S2). On the other hand, *Cbln1* heterozygotes showed more than half of CBLN1 protein loss in the cerebellum but displayed no markedly impaired performance on the accelerating rotarod test [6]. *CbIn1* heterozygous mice might have developed some compensatory mechanisms, while *Synage* KO- or knockdown-induced 50% *Cbln1* downregulation may be sufficient to manifest the phenotype of synapses loss. In addition, *Cbln1* was also found to mediate specific aspects of fear conditioning and spatial memory differentially and regulate both motor and non-motor functions [63]. We thus speculated that both *Synage* and *Cbln1* likely have additional functions that are independent of one another. *Synage* probably modulates cerebellar development and function through other mechanisms in addition to regulation of *Cbln1* expression.

### *Synage* lncRNA modulates cerebellar synapses by orchestrating assembly of synaptic LRP1-HSP90AA1-PSD-95 complex

**Fig. 5:**
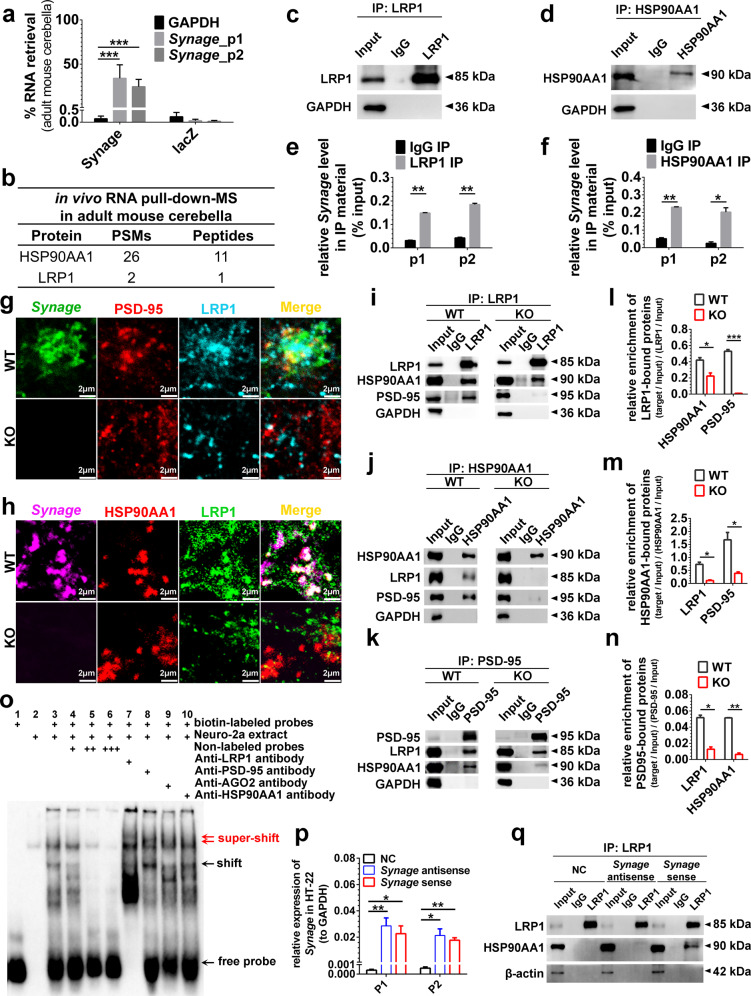
*Synage* lncRNA acts as an organizer to scaffold the synaptic LRP1-HSP90AA1-PSD-95 complex. **a** RT-qPCR analysis of in vivo RNA pull-down showing retrieval of *Synage* lncRNA with *Synage*-specific probes in the adult mouse cerebella. LacZ probes were the negative controls. *Synage*_p1 and *Synage*_p2 represent different primers targeting *Synage* (all three isoforms). **b** List of top *Synage*-binding proteins detected in the adult mouse cerebella by in vivo RNA pull-down–mass spectrometry. **c–f** Western blots assessing LRP1 and HSP90AA1 immunoprecipitation (IP) by anti-LRP1 (**c**) or anti-HSP90AA1 (**d**) antibody in RNA immunoprecipitation (RIP). Relative enrichment of *Synage* binding to LRP1 (**e**) or HSP90AA1 (**f**) or IgG in IP was quantified as a percentage of input by RT-qPCR. p1 and p2 represent different primers targeting *Synage* (all three isoforms). **g**, **h** Co-localization analysis of *Synage* with PSD-95 and LRP1 (**g**) as well as with HSP90AA1 and LRP1 (**h**) by RNA-FISH of *Synage* and immunofluorescence staining of these proteins in the 2-month-old WT and KO mouse cerebella. **i**–**n** Western blots and relative quantification of the interactions among LRP1 (**i**, **l**), HSP90AA1 (**j**, **m**), and PSD-95 (**k**, **n**) as determined by co-IP analysis in the adult WT and KO mouse cerebella. **o** RNA-electrophoretic mobility shift assays (EMSAs) for verification of the direct interactions between *Synage* and its binding proteins in the Neuro-2a cell line. **p**, **q** RT-qPCR analysis of the overexpression level of *Synage* (sense) in the HT-22 cell line (**p**), Western blots assessing LRP1 and HSP90AA1 co-immunoprecipitation by anti-LRP1 antibody after *Synage* overexpression in the HT-22 cell line, *Synage* antisense RNA was used as a negative control (**q**).

In our in vivo RNA pull-down–MS experiments, LRP1 and HSP90AA1 were the two strongest candidates identified by MS in the cerebellum (Fig. 5a, b). We conducted LRP1-RIP and HSP90AA1-RIP assays (Fig. 5c, d), and *Synage* was detected in both LRP1-RIP and HSP90AA1-RIP samples (Fig. 5e, f), confirming that *Synage* bound both LRP1 and HSP90AA1 and might form a complex. LRP1 is known to interact with PSD-95, through which it modulates synaptic function [9, 64]. We noted a similarity of the neuronal loss in mice induced by LRP1 deletion and the *Synage* KO (Supplementary Table S2). We thus speculated that PSD-95 may also be in the complex of LRP1-HSP90AA1-*Synage*. RNA-FISH in combination with IF using different fluorophores to label *Synage*, LRP1, PSD-95, and HSP90AA1, showed co-localization of *Synage* with all three proteins in PCs of WT cerebella, and the co-localization was significantly reduced in PCs of *Synage* KO cerebella (Fig. 5g, h).

We also examined the influence of *Synage* on LRP1-HSP90AA1-PSD-95 interactions by performing co-IP with mouse cerebellum tissue (Fig. 5i–k) and found that the interactions among all three of these proteins were reduced in *Synage* KO mice compared with WT controls (Fig. 5l–n). Although IF experiments revealed a decrease in the protein level of LRP1 in the cerebellar cortex of KO mice compared to WT mice (Figs. 5g, h and  S9a), the total protein levels of the LRP1, HSP90AA1, and PSD-95 did not differ when examined by Western blots of cerebella samples of adult WT versus *Synage* KO mice (Fig. S9b, c). These data suggest that *Synage* functions in synapse stability not by reducing protein levels per se, but rather by somehow regulating *Synage*-dependent assembly of the LRP1-HSP90AA1-PSD-95 complex in the cerebellar cortex.

We further investigated the possibility of interactions between *Synage* and each of these proteins (LRP1, PSD-95, and HSP90AA1) using biotin-labeled n424059 (one of the *Synage* isoforms) sense probes in the Neuro-2a cell line by EMSAs (Fig. 5o). The Neuro-2a cell line is a fast-growing mouse neuroblastoma cell line [65, 66], and n424059 is not expressed in this cell line, so that we could exclude the interference from endogenous n424059 (Fig. S10). The EMSAs data revealed that LRP1 and PSD-95 could each bind to *Synage* (Fig. 5o). Furthermore, overexpression of *Synage* in the HT-22 cell line significantly increased the interaction between LRP1 and HSP90AA1 compared with the controls, as shown by RT-qPCR and co-IP experiments (Fig. 5p, q).

In addition, we knocked down each protein (LRP1, HSP90AA1, and PSD-95) by three shRNAs specifically targeting their mRNAs respectively in the HT-22 cell line (Fig. S11a–c). PSD-95 protein was dramatically reduced upon knockdown of LRP1 or HSP90AA1 in the HT-22 cell line (Fig. S11a, b, d, e). Furthermore, the LRP1 protein was significantly upregulated upon knockdown of HSP90AA1 or PSD-95 (Fig. S11b, c, e, f). These results demonstrated that knocking down LRP1 or HSP90AA1 or PSD-95 impacted the levels of the other two proteins comprising the LRP1-HSP90AA1-PSD-95 complex. We also found that, after LRP1 knockdown, overexpression of *Synage* in the HT-22 cell line significantly increased the extent of the LRP1-HSP90AA1 interaction as compared to the random shRNA control (Fig. S11g, h), indicating that LRP1 depletion inhibits the interactions between LRP1-HSP90AA1 and *Synage*. Together, these results demonstrate the function of *Synage* as a key organizer of the LRP1-HSP90AA1-PSD-95 complex in PCs, to maintain the stability and function of these proteins in cerebellar synapses.

## Discussion

**Fig. 6:**
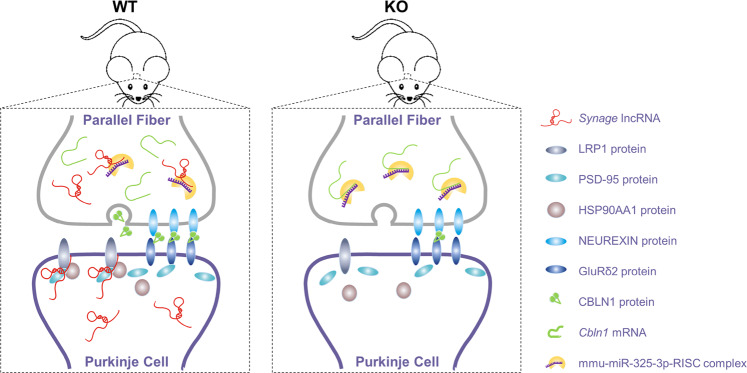
A schematic diagram of the mechanism of the *Synage* lncRNA in the regulation of synaptic and neuronal function in the WT and *Synage* KO mouse cerebella. *Synage* lncRNA regulates stability and function of cerebellar synapses via at least two mechanisms. One is through the function of *Synage* as a sponge for mmu-miR-325-3p to regulate *Cbln1* mRNA expression, which leads to the change of the CBLN1 protein levels. The other function is to serve as a scaffold for orchestrating the assembly of synaptic LRP1-HSP90AA1-PSD-95 complex.

lncRNAs are abundant in the brain. In the present work, we discovered that a cerebellum highly expressed lncRNA, *Synage*, mainly distributed in the cytoplasm and extending into neurites and synapses, regulate cerebellar synaptic stability and cerebellar development starting at the E12.5 embryo stage. *Synage* KO leads to a dramatic decrease in cerebellar neuron number, synapse density, and synaptic stability in mice cerebella. At the molecular level, we showed that *Synage* regulates synaptic stability and function by *Synage*-dependent assembly of the LRP1-HSP90AA1-PSD-95 complex in the cerebellar cortex (Fig. 6). Both HSP90AA1 and LRP1 have been reported to exhibit anti-apoptotic effects in neurons by directly binding and/or activating Akt kinase [67, 68]. We thus speculate that the interaction of *Synage* with HSP90AA1 and LRP1 might increase Akt activation and thus protect PC and GC neurons from apoptosis in the WT mice. In addition, *Synage* appears to be multitasking in that it also functions as a ceRNA to adsorb a *Cbln1*-targeted miRNA to upregulate *Cbln1* expression in GCs (Fig. 6). *Synage* KO mice were more severely impaired than the *Cbln1*^−/−^ mice (Supplementary Table S2), consistent with our data showing that *Synage* exerts its function via at least two mechanisms, first by regulating the expression level of *Cbln1* (in GCs), and second by orchestrating the formation of the synaptic LRP1-HSP90AA1-PSD-95 complex (in PCs).

The synapses between climbing fibers and PCs, as well as those between PFs and PCs, are known to form in the cerebellar cortex after birth [69–71]. Our data, however, showed that the loss of PCs occurred during embryonic development of the *Synage* KO cerebellum. Accordingly, our data support that the observed decrease in PCs number in the *Synage* KO mice during embryonic development clearly has longer term impacts that manifest later as reductions in synapse density and in synaptic activity after birth. Although we cannot rule out the possibility that the reduced PCs number may also affect synaptic formation or maintenance, our data indicate that altered synaptic formation (or maintenance) after birth is impacted by the destruction of *Synage*-binding protein complexes in the *Synage* KO cerebellum.

In summary, this study demonstrates that cerebellum highly expressed *Synage* lncRNA regulates the formation of the LRP1-HSP90AA1-PSD-95 complex in PCs and *Cbln1* expression in GCs. These interacting components are located in synapses where they perform unique roles, thereby differentially affecting synaptic stability and functionality, which in turn affects the growth of neurons, the development and function of the cerebellum, and greatly contributes to motor function during development. Since the genomic localization and the cerebellar distribution of *Synage* are conserved from mice to humans, targeting of the LRP1-HSP90AA1-PSD-95-*Synage* complex or *Synage*-*Cbln1* interactions may protect against developmental defects in humans and in model organisms; insights into their neuroprotective mechanisms can potentially yield pharmaceuticals designed to extend neurological health and synaptic function, and to mitigate cerebellar neurodevelopmental disorders.

## Supplementary information

Supplementary Materials

## Data Availability

The accession number for the ribo-minus RNA-Seq data reported in this paper is CRA001819 and is publicly accessible at https://bigd.big.ac.cn/gsa.
