# Supplementary material for: The long noncoding RNA Synage regulates synapse stability and neuronal function in the cerebellum
Source: Cell Death Differ. 2021 Mar 24;28(9):2634–50. doi: 10.1038/s41418-021-00774-3 (PMC8408218; doi:10.1038/s41418-021-00774-3)
Supplement: Supplementary file 1 — Supplementary Materials [file 41418_2021_774_MOESM1_ESM.pdf]

Supplemental Data for

The long noncoding RNA *Synage* regulates synapse stability and neuronal function in the cerebellum

Fei Wang[†], Qianqian Wang[†], Baowei Liu[†], Lisheng Mei, Sisi Ma, Shujuan Wang, Ruoyu Wang, Yan Zhang, Chaoshi Niu, Zhiqi Xiong, Yong Zheng, Zhi Zhang, Juan Shi, and Xiaoyuan Song*

[†]These authors contributed equally to this work.
*Corresponding author. Email: songxy5@ustc.edu.cn

This PDF file includes:

Supplemental Figures S1-S11
Supplemental Tables S1-S3

Supplemental Figure S1 (related to Figure 1)

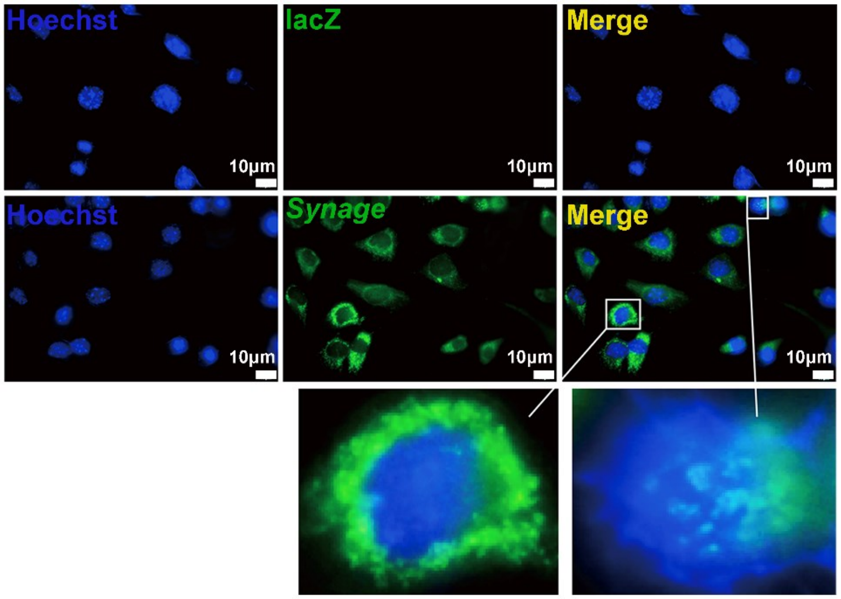

Fig. S1 Distribution of *Synage* lncRNA in the C8-D1A cell line. RNA FISH of *Synage* (green) in the C8-D1A cerebellum cell line, lacZ probe (green) was used as a negative control. Nuclei were stained with Hoechst 33342 (blue). Scale bar: 10 μm.

Supplemental Figure S2 (related to Figure 1)

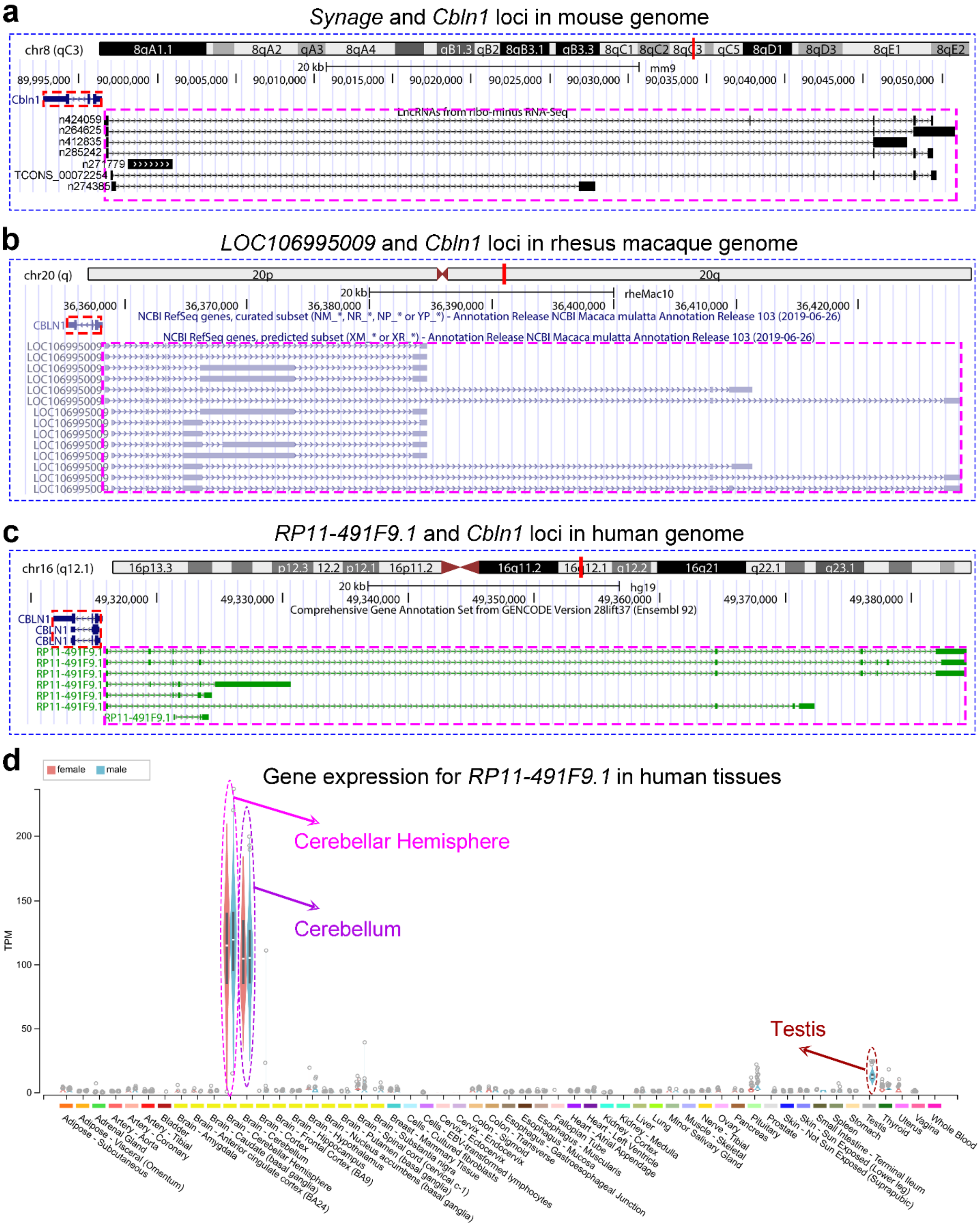

Fig. S2 *Synage* lncRNA is conserved in its genomic location and in its distribution specificity in the cerebellum. a Schematic view of the genomic loci of the *Synage* transcripts in the mouse brains detected by ribo-minus RNA-Seq. **b and c** Schematic view of the genomic loci of the homologous *Synage*, *LOC106995009* and *RP11-491F9.1*, in the rhesus macaque **(b)** and human genomes **(c)**, respectively. The *Cbln1* gene is shown in the red box. The genomic loci of both *Synage* in the mouse and homologous *Synage* in the rhesus macaque and human are shown in the purple box. **d** Gene expression level for *RP11-491F9.1* in human tissues screened from the GTEx project database (dbGaP Accession phs000424.v8.p2).

Supplemental Figure S3 (related to Figure 1)

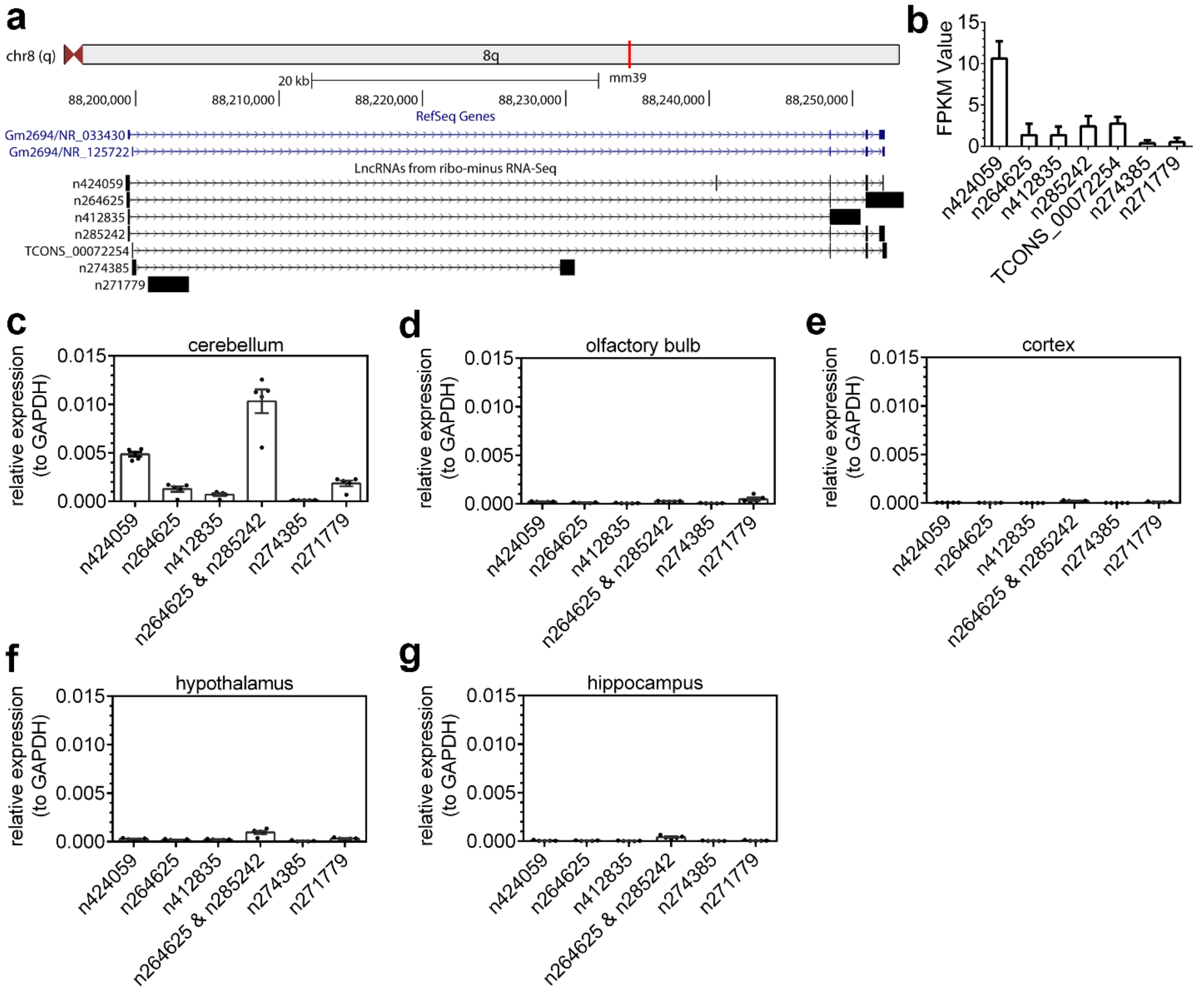

Fig. S3 The expression level of the mouse *Synage* transcripts in different brain regions. a Schematic view of *Synage* transcripts detected by ribo-minus RNA-Seq. **b** FPKM (Fragments Per Kilobase Million) value of the *Synage* transcripts in the whole brains of 2-month-old mice detected by ribo-minus RNA-Seq (n=3). **c-g** The relative expression level of the *Synage* transcripts in the adult mouse cerebellum **(c)**, olfactory bulb **(d)**, cortex **(e)**, hypothalamus **(f)**, and hippocampus **(g)**, detected by RT-qPCR (n=5). The sequence of n264625 overlaps with most of the sequence of n285242, so that a RT-qPCR primer pair specifically targeting n285242 was unable to be designed, but a RT-qPCR primer pair specifically targeting both n285242 and n264625 can be designed, so does a RT-qPCR primer pair specifically targeting n264625. The expression level of n285242 was estimated indirectly by removing the expression level of n264625 transcript from the total expression level of n285242 and n264625 transcripts detected by RT-qPCR.

Supplemental Figure S4 (related to Figure 1)

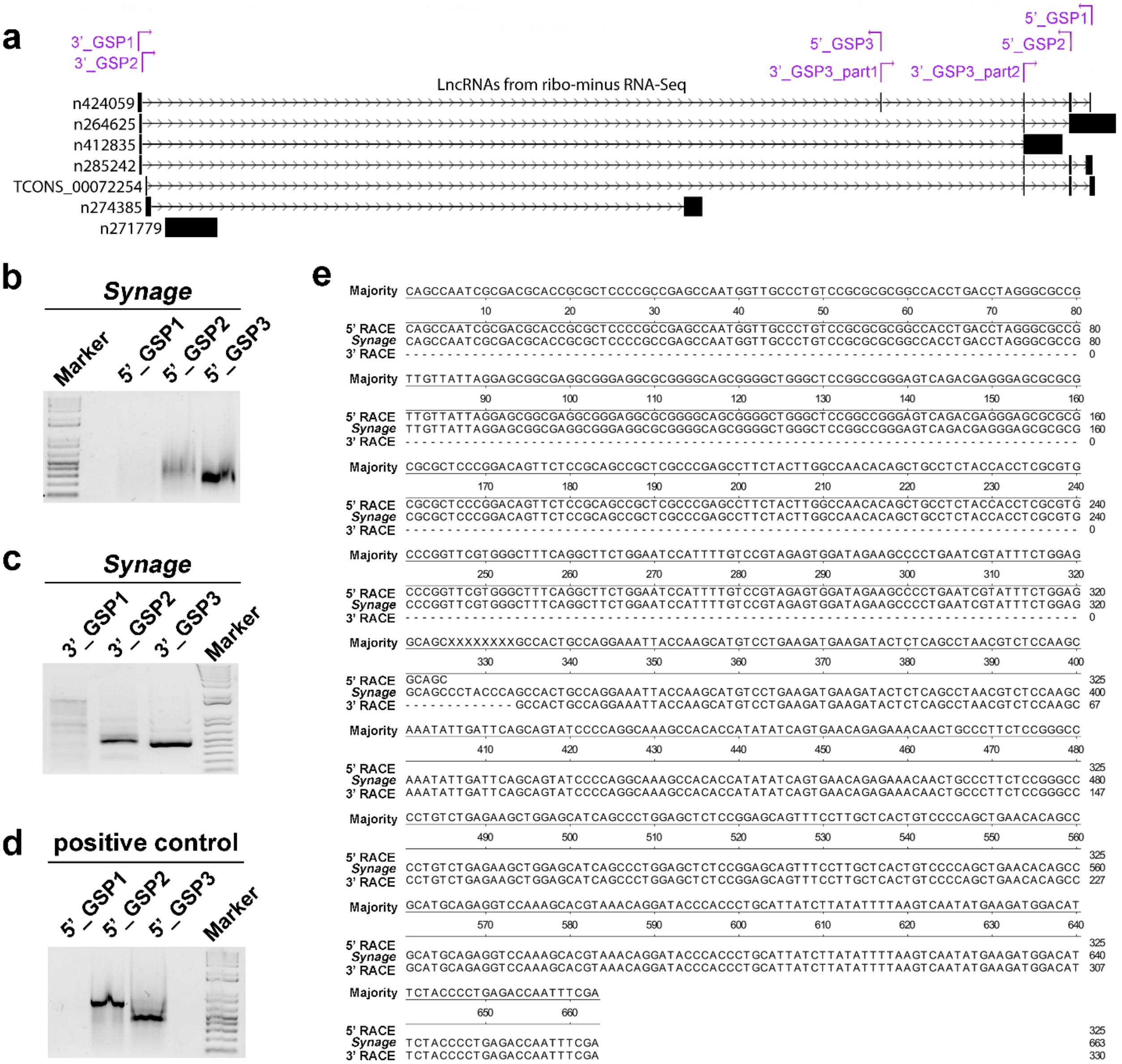

Fig. S4 The characterization of the full length mouse *Synage* lncRNA. a The position of gene specific primers (GSPs) targeting *Synage* in the 5'- and 3'-RACE experiments. **b-d** The 5'- **(b)** and 3'-RACE **(c)** PCR amplification of *Synage* fragments using respective GSPs. The 4.8 kb pAMP1 5'-RACE recombinant was a positive control, which was provided by the commercial 5'-RACE kit **(d)**. **e** cDNA sequencing and assembling of 5'- and 3'-RACE of *Synage* fragments.

Supplemental Figure S5 (related to Figure 2)

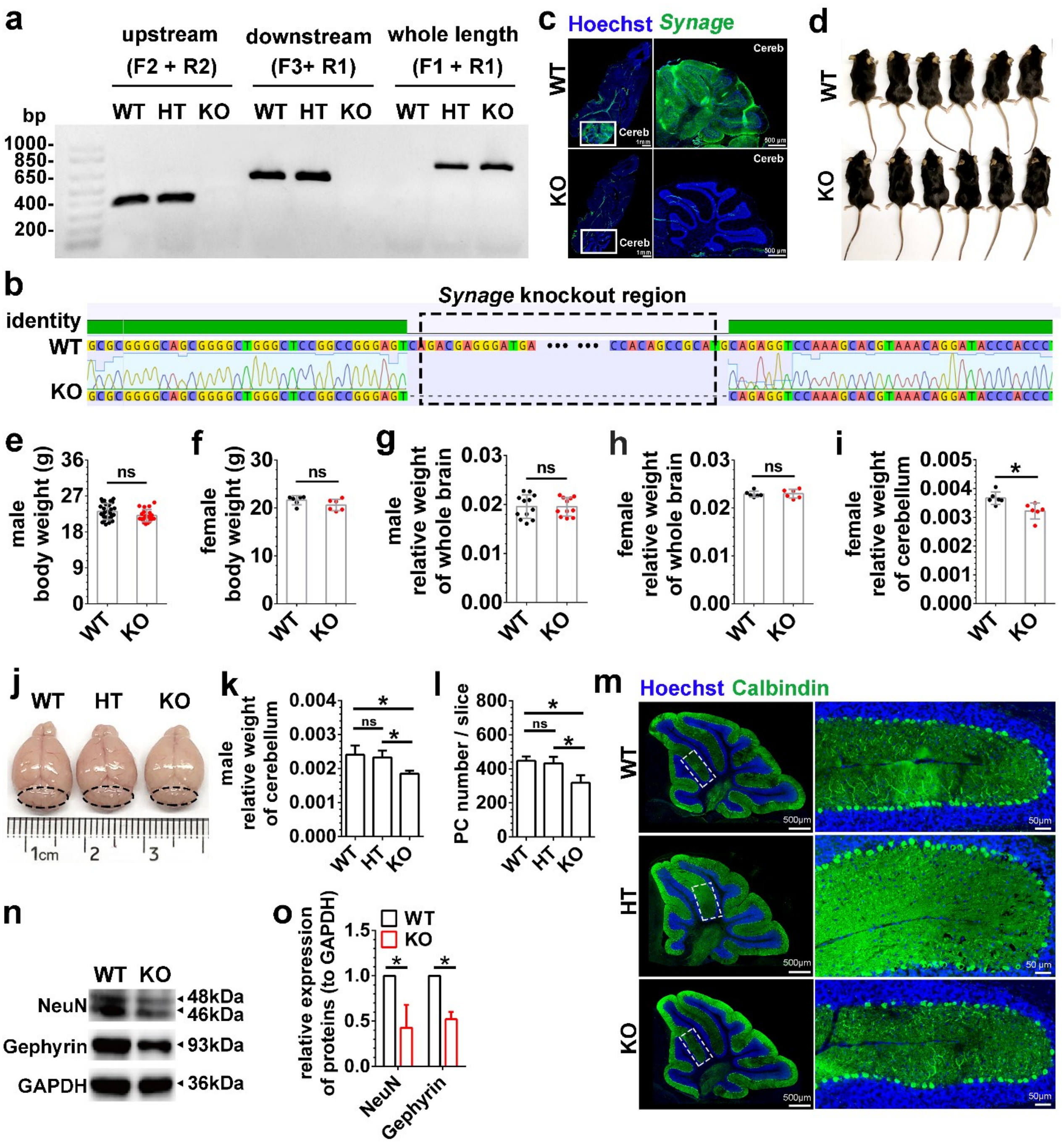

Fig. S5 *Synage* knockout causes cerebellar atrophy and neuronal loss. a Genotype identification of *Synage* wild-type (WT), heterozygous (HT), and homozygous (KO) mice by genomic PCR amplification for *Synage*. **b** Genomic sequencing of *Synage* in WT and *Synage* KO mice. **c** The distribution of *Synage* (green) in the brain sections of WT and KO adult mice detected by RNA FISH. Nuclei were stained with Hoechst 33342 (blue). Left scale bar: 1 mm; right scale bar: 500 μm. **d** Representative images of 2-month-old WT and KO male mice. **e and f** Body weights of adult WT and KO male **(e)** and female **(f)** mice. **g and h** Relative weights of whole brain in adult male **(g)** and female **(h)** mice. **i** Relative weights of the cerebella in adult female mice, normalized to body weight, each dot represents a mouse. **j and k** Representative images **(j)** and relative weights **(k)** of the cerebella of 2-month-old WT, *Synage* HT, and KO male mice, normalized to body weight (n=3). **l and m** Quantification of the number of Purkinje cells per cerebellar sections **(l)** and representative immunofluorescence staining images of Purkinje cells **(m)** in the cerebella of adult WT, *Synage* HT, and KO male mice (n=3). Nuclei were stained with Hoechst 33342 (blue). Left scale bar: 500 μm; right scale bar: 50 μm. **n and o** Representative images of Western blots **(n)** and quantification **(o)** for both NeuN (a neuronal marker) and Gephyrin (an inhibitory postsynaptic marker) in adult WT and KO mouse cerebella, normalized to GAPDH.

Supplemental Figure S6 (related to Figure 2)

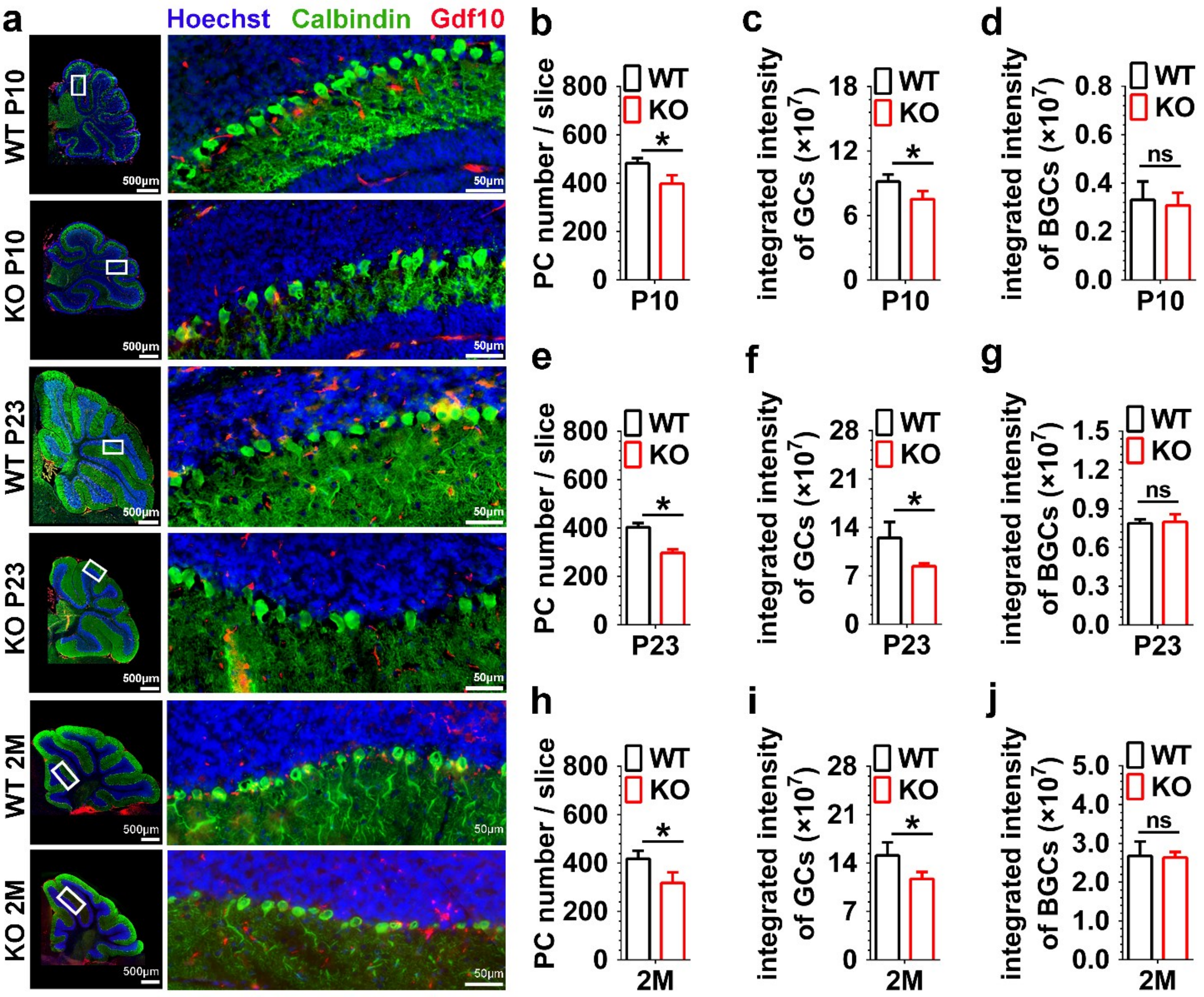

Fig. S6 ***Synage*** **knockout mice show neuronal loss during cerebellar development. a-j** Representative immunofluorescence staining images **(a)** and quantification of the number of PCs (Calbindin, green), the integrated intensity of granule cells (GCs), and the integrated intensity of Bergmann glial cells (BGCs, Gdf10, red) in the cerebella from P10 **(b-d)**, P23 **(e-g)**, and 2-month-old **(h-j)** WT and *Synage* KO mice. Nuclei were stained with Hoechst 33342 (blue). Left scale bar: 500 μm; right scale bar: 50 μm. The signal intensity of Hoechst 33342 staining in the granule cell layer was used to estimate GCs.

Supplemental Figure S7 (related to Figure 3)

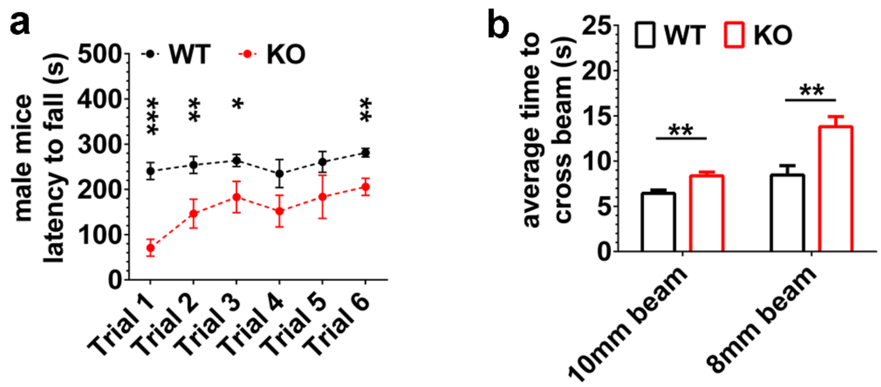

Fig. S7 *Synage* knockout impairs motor abilities and motor-dependent learning and memory of mice. a The time(s) that adult mice stayed on the rotarod when tested at constant speeds between 4 and 40 rpm before falling in the rotarod test (WT: n = 10; KO: n = 8). **b** The average time(s) that it took adult mice to cross an 80 cm beam with a flat surface of 10 mm or 8 mm width in the balance beam test (WT: n = 12; KO: n = 12).

Supplemental Figure S8 (related to Figure 4)

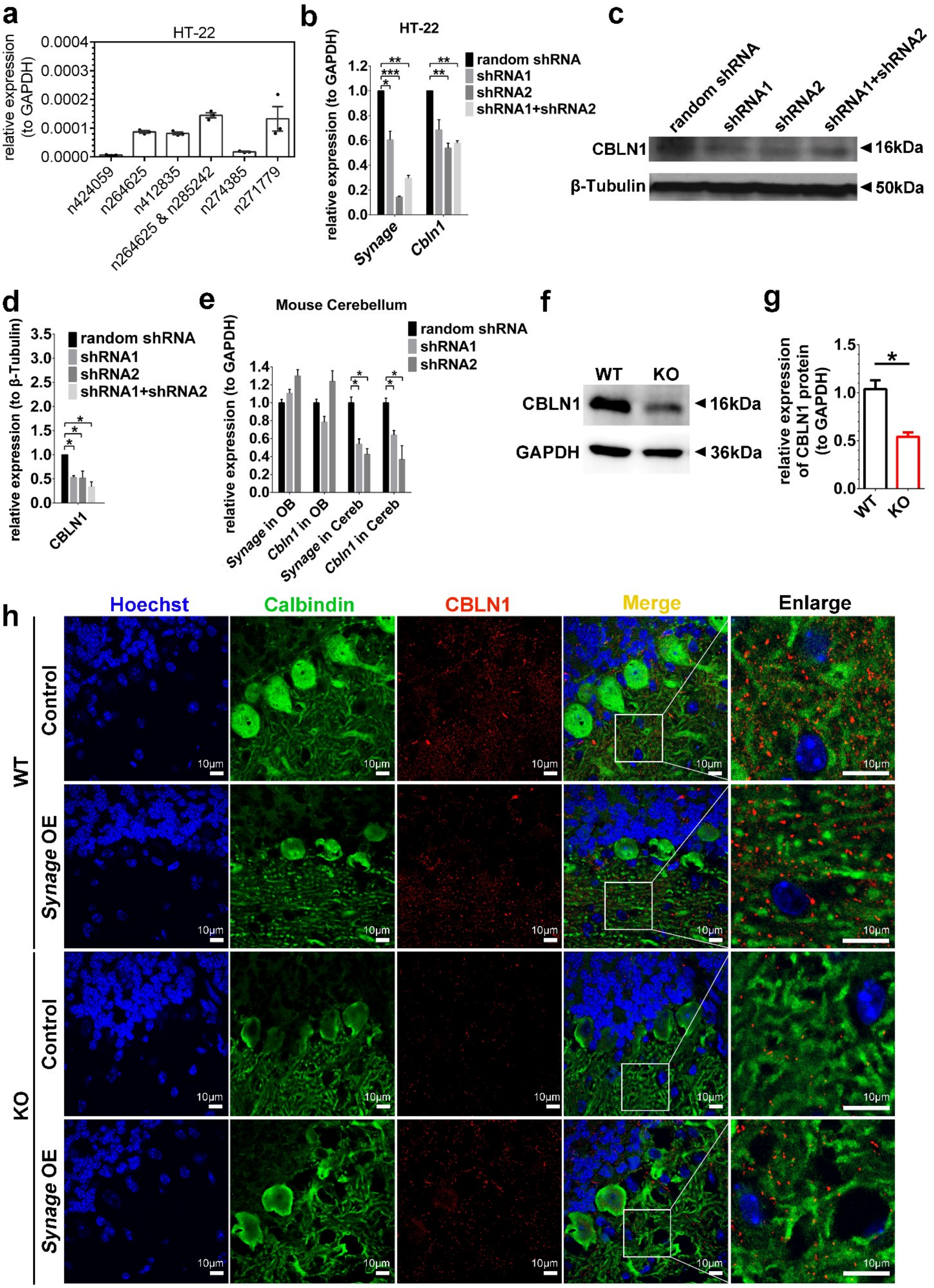

Fig. S8 Knockdown of *Synage* results in substantial reduction in the expression of *Cbln1 in vitro* and *in vivo*. **a** Relative expression levels of *Synage* transcripts in the HT-22 cell line detected by RT-qPCR. **b** Relative expression levels of *Synage* lncRNA and *Cbln1* mRNA after *Synage* shRNA knockdown in the HT-22 cell line detected by RT-qPCR. **c and d** Western blot analysis **(c)** and quantification **(d)** for CBLN1 protein in the HT-22 cell line following *Synage* knockdown, normalized to β-Tubulin. **e** Relative expression levels of *Synage* lncRNA and *Cbln1* mRNA after *Synage* shRNA knockdown in the mouse cerebella detected by RT-qPCR (OB, olfactory bulb; Cereb, cerebellum). **f and g** Representative images of Western blot **(f)** and quantified expression level **(g)** of CBLN1 protein, normalized to GAPDH, in 2-month-old WT and KO mice. **h** Representative confocal images of CBLN1 with Calbindin by immunofluorescence staining in 3-week-old WT and KO mouse cerebella after stereotaxic injection of AAV-control or AAV-*Synage* into the neonatal mouse cerebella. Scale bar: 10 μm.

Supplemental Figure S9 (related to Figure 5)

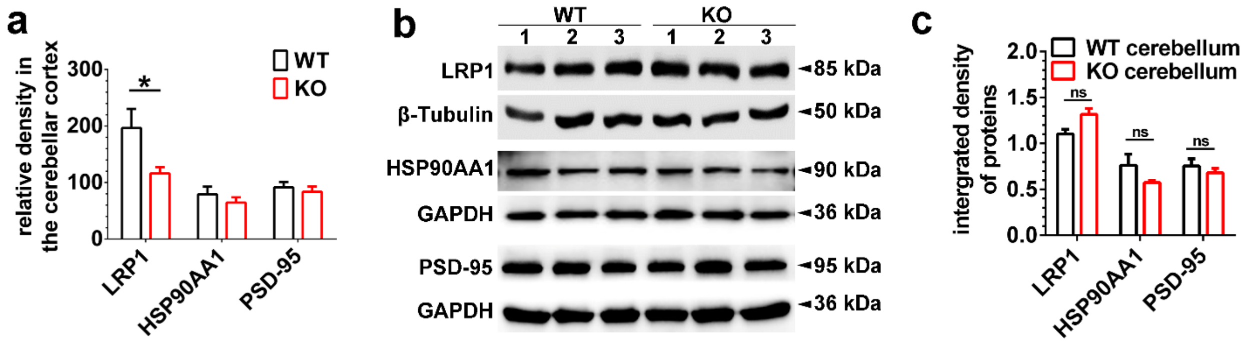

Fig. S9 Identification and verification of *Synage*-binding proteins. a Relative quantification of LRP1, HSP90AA1, and PSD-95 in the cerebellar cortex of 2-month-old WT and KO mice detected by immunofluorescence staining of the related proteins. **b-c** Western blots **(b)** and relative quantification **(c)** for LRP1, HSP90AA1, and PSD-95 proteins in the WT and KO mouse cerebella (n=3).

Supplemental Figure S10 (related to Figure 5)

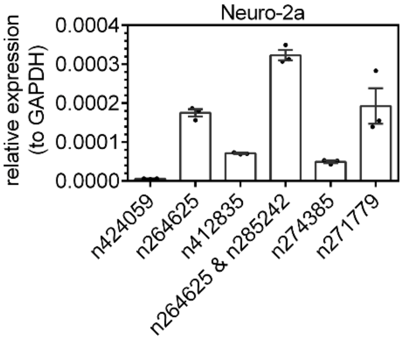

Fig. S10 Relative expression levels of *Synage* transcripts in the Neuro-2a cell line detected by RT-qPCR.

Supplemental Figure S11 (related to Figure 5)

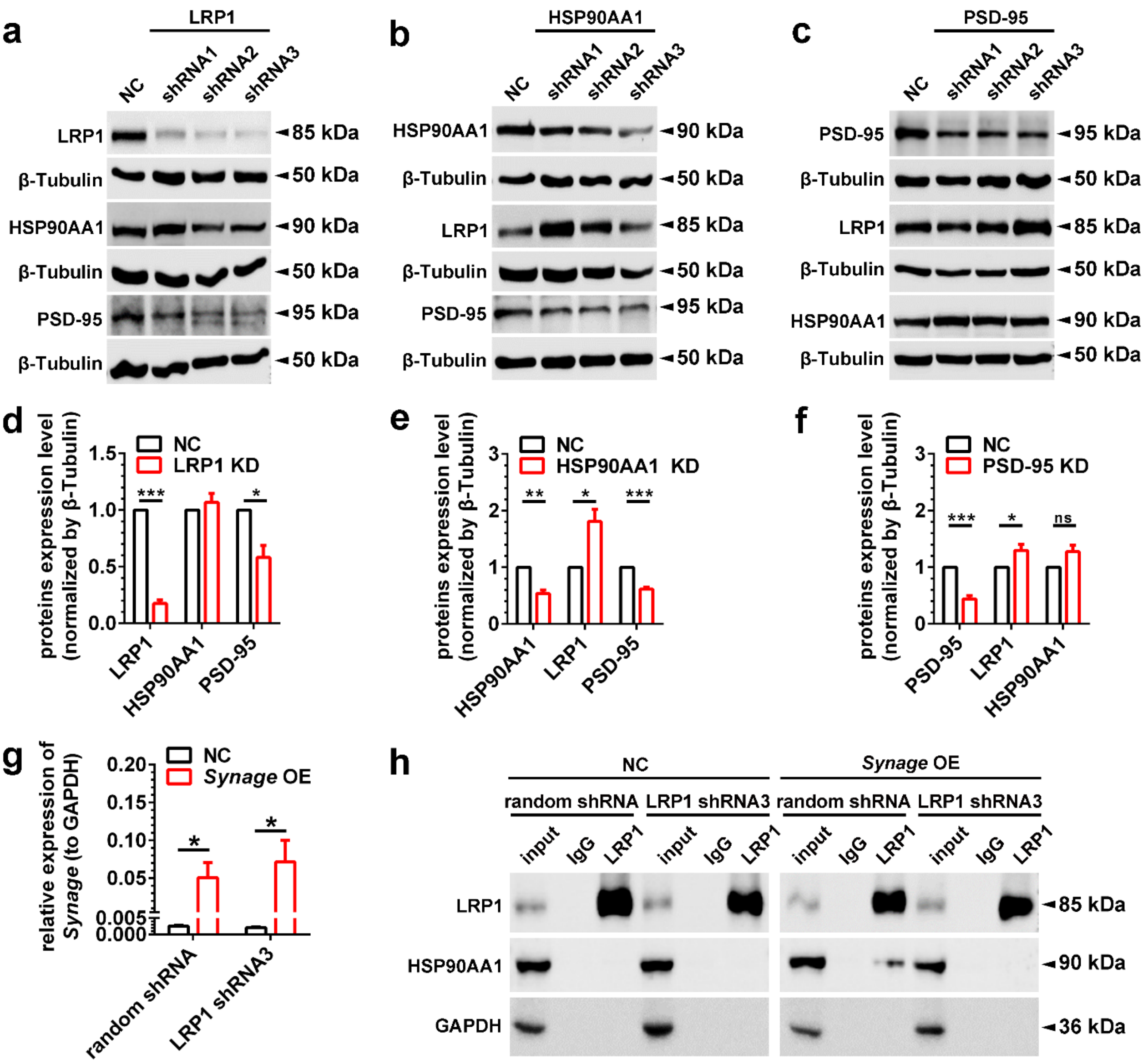

Fig. S11 LRP1 depletion inhibits the interaction between LRP1-HSP90AA1 and *Synage*. a-f Western blots **(a-c)** and quantification **(d-f)** for knockdown analyses of LRP1 (**a, d**), HSP90AA1 (**b, e**), and PSD-95 (**c, f**) protein in the HT-22 cell line following each protein knockdown, normalized to β-Tubulin. **g** The expression level of *Synage* lncRNA after LRP1 knockdown and *Synage* overexpression in the HT-22 cell line detected by RT-qPCR. **h** Western blots assessing LRP1 and HSP90AA1 immunoprecipitation (IP) by anti-LRP1 antibody after LRP1 knockdown and *Synage* overexpression in the HT-22 cell line.

Supplemental Table S1. AGO2 CLIP-Seq peak information in mouse cortex

**AGO2 CLIP-Seq peak information in mouse cortex (screened from GSE73058, Moore et al., 2015)**					
Chromosome	Strand	Start	End	Peak.ID	Gene symbol
	-	89,992,794	89,992,854	200144	*Cbln1*
	-	89,992,926	89,992,986	200161	*Cbln1*
	-	89,993,134	89,993,194	200186	*Cbln1*
	-	89,993,267	89,993,327	200198	*Cbln1*
	-	89,993,327	89,993,387	200212	*Cbln1*
	-	89,993,503	89,993,563	200243	*Cbln1*
	-	89,993,797	89,993,857	200292	*Cbln1*
	-	89,993,904	89,993,964	200314	*Cbln1*
chr8	-	89,994,005	89,994,065	200330	*Cbln1*
	-	89,994,208	89,994,268	200347	*Cbln1*
	-	89,995,646	89,995,706	200385	*Cbln1*
	-	89,995,883	89,995,943	200408	*Cbln1*
	-	89,996,015	89,996,075	200437	*Cbln1*
	+	90,016,211	90,016,271	142479	*Synage* (*Gm2694*)
	+	90,022,615	90,022,675	142568	*Synage* (*Gm2694*)
	+	90,028,588	90,028,648	142659	*Synage* (*Gm2694*)
	+	90,038,866	90,038,926	142745	*Synage* (*Gm2694*)

Supplemental Table S2. Phenotypes of knockout mice

**Phenotypes**	***Synage***$^{-/-}$ **mice**	***Cbln1***$^{-/-}$ **mice**[(1-3)]	***LRP1***$^{-/-}$ **mice**[(4, 5)]
Decreased synaptic vesicles	+	-	NA
Impaired synaptic function	+	+	+
Synaptic loss	+	+	+
Neuronal loss	+	-	+
Abnormal electrophysiology	+	+	+
Motor dysfunction and memory deficits	+	+	+
Reduced fertility	+	-	+
Decreased cerebellar weight	+	-	NA

Abbreviations: +, present; -, absent; NA, not applicable

Supplemental Table S3. sgRNA/shRNA and primer sequences

Name	Application	Sequence (5'-3')
*Synage*_sgRNA 1	CRISPR/Cas9	TCCGGCCGGGAGTCAGACGA
*Synage*_sgRNA 2	CRISPR/Cas9	AAGGGCATGGTGGGTTGGCG
*Synage*_genotype_F1	genotype	GAGCCAATGGTTGCCCTGTC
*Synage*_genotype_F2	genotype	ACAAAGGCGCGGATCAAGC
*Synage*_genotype_F3	genotype	GGATACCCACCCTGCATTATC
*Synage*_genotype_R1	genotype	AACTCCACTGTTACTGCTAATACA
*Synage*_genotype_R2	genotype	CTCCTACCTGAAAGCCCACGAA
*GAPDH*_F	RT_qPCR	ACATCATCCCTGCATCCACTG
*GAPDH*_R	RT_qPCR	CCTGCTTCACCACCTTCTTG
*Synage*_p1_F	RT_qPCR	ACCAAGCATGTCCTGAAGATG
*Synage*_p1_R	RT_qPCR	CTGGGGATACTGCTGAATCAA
*Synage*_p2_F	RT_qPCR	ACTCTCAGCCTAACGTCTCCAA
*Synage*_p2_R	RT_qPCR	GATGCTCCAGCTTCTCAGACAG
*Cbln1*_F	RT_qPCR	CTGGCTGTATTCCGTATT
*Cbln1*_R	RT_qPCR	ACAAGCATCAGAGAACAA
mmu-mir-325-3p_F	RT_qPCR	TTAGCGTTAGCGTTTATTGAGCAC
mmu-mir-325-3p_R	RT_qPCR	TATGGTTGTTCACGAGTCCTTGTC
*U6*_F	RT_qPCR	ATTGGAACGATACAGAGAAGATT
*U6*_R	RT_qPCR	GGAACGCTTCACGAATTTG
n424059_F	RT_qPCR	TCGCCCGAGCCTTCTACTTG
n424059_R	RT_qPCR	CAGGGGCTTCTATCCACTCTAC
n264625 (*Synage*-P1)_F	RT_qPCR	GATTCAGCTCGCTCACACCT
n264625 (*Synage*-P1)_R	RT_qPCR	CATTGTGCATGTAGCAGCGAA
n412835_F	RT_qPCR	TGCTGATAACCCACCTTGGC
n412835_R	RT_qPCR	AAGACCGCACCACAATCCAT
n264625 & n285242 (*Synage*-P2)_F	RT_qPCR	AGAGAAACAACTGCCCTTCTCC
n264625 & n285242 (*Synage*-P2)_R	RT_qPCR	TTCCTGAGCAGGCAGTATCC
n274385_F	RT_qPCR	GTGAAACTACATCCACGCCCA
n274385_R	RT_qPCR	CTGACCGTTGTTCTAACGCA
n271779_F	RT_qPCR	ACGTACCCTGCACACACATA
n271779_R	RT_qPCR	GCCAAAGCCCCCAGCTAATA
*Cbln1*_NRO_F1	NRO	GTCTACAACAGACAGACCATCC
*Cbln1*_NRO_R1	NRO	CACTGATTTCTGACGCTTG
*Cbln1*_NRO_F2	NRO	ATCAGGAGCACCAACCATGA
*Cbln1*_NRO_R2	NRO	CAATCCATTCTGGGTGCTGC
*Gm2694*_NRO_F1	NRO	AACACAGCTGCCTCTACCAC
*Gm2694*_NRO_R1	NRO	TCGGCGCGATGCTGCACT
*Gm2694*_NRO_F2	NRO	AGTGCTGCTTGTTCGAACCCAT
*Gm2694*_NRO_R2	NRO	TCTTCATCTTCAGGACATGC
*Gm2694*_NRO_F3	NRO	TGTCTGAGAAGCTGGAGCATCA
*Gm2694*_NRO_R3	NRO	GAACGGGAGTAGATCTGAC
*HPRT*_NRO_F1	NRO	GCTTCCTCCTCAGACCGCTT
*HPRT*_NRO_R1	NRO	TCTGCTGGAGTCCCCTTG
*HPRT*_NRO_F2	NRO	GGAATGTGTCCTGTAAAAGT
*HPRT*_NRO_R2	NRO	CTCCCATCTCCTTCATGACA
*HPRT*_NRO_F3	NRO	CAGACAACGTAGGAGGAC
*HPRT*_NRO_R3	NRO	CCAGTTTCACTAATGACAC
*Synage*_3'RACE_GSP1	RACE	CAGCCAATCGCGACGCAC
*Synage*_3'RACE_GSP2	RACE	GGCCAACACAGCTGCCTCTAC
*Synage*_3'RACE_GSP3	RACE	GCCACTGCCAGGAAATTACCAAG
*Synage*_5'RACE_GSP1	RACE	TCGAAATTGGTCTCAGGGGT
*Synage*_5'RACE_GSP2	RACE	CTGATGCTCCAGCTTCTCAGACAG
*Synage*_5'RACE_GSP3	RACE	GCTGCCTCCAGAAATACGATTCAG
*Synage*_shRNA1	Knockdown	TCCAGAAATACGATTCAGG
*Synage*_shRNA2	Knockdown	AGCGCGGTGCGTCGCGATT

*LRP1*_shRNA1	Knockdown	TTACCAAAGAATGTGTTCAGC
*LRP1*_shRNA2	Knockdown	TTAGCCAGTGTATTTGTTCGC
*LRP1*_shRNA3	Knockdown	TTCATACATCTTGTAGGTAGG
*HSP90AA1*_shRNA1	Knockdown	AAACTTAGGGTTGTTCTCGGG
*HSP90AA1*_shRNA2	Knockdown	ATCATCCTCATCAATACCTAG
*HSP90AA1*_shRNA3	Knockdown	AATGAAATTCAGATACTCAGG
*PSD-95*_shRNA1	Knockdown	TATACTGAGCGATGATCGTG
*PSD-95*_shRNA2	Knockdown	ACACTGTTGACCGCCAGGATCTTGTCTCC
*PSD-95*_shRNA3	Knockdown	GAGTCTCTCTCGGGCTGGGACCCAGATGT

References

1. Uemura T, Lee SJ, Yasumura M, Takeuchi T, Yoshida T, Ra M, et al. Trans-synaptic interaction of GluRdelta2 and Neurexin through Cbln1 mediates synapse formation in the cerebellum. Cell. 2010;141(6):1068-79.
2. Rong Y, Wei P, Parris J, Guo H, Pattarini R, Correia K, et al. Comparison of Cbln1 and Cbln2 functions using transgenic and knockout mice. J Neurochem. 2012;120(4):528-40.
3. Hirai H, Pang Z, Bao D, Miyazaki T, Li L, Miura E, et al. Cbln1 is essential for synaptic integrity and plasticity in the cerebellum. Nat Neurosci. 2005;8(11):1534-41.
4. Liu Q, Trotter J, Zhang J, Peters MM, Cheng H, Bao J, et al. Neuronal LRP1 knockout in adult mice leads to impaired brain lipid metabolism and progressive, age-dependent synapse loss and neurodegeneration. J Neurosci. 2010;30(50):17068-78.
5. May P, Rohlmann A, Bock HH, Zurhove K, Marth JD, Schomburg ED, et al. Neuronal LRP1 functionally associates with postsynaptic proteins and is required for normal motor function in mice. Mol Cell Biol. 2004;24(20):8872-83.
